# Comparative Phenotypic Analysis of the Major Fungal Pathogens *Candida parapsilosis* and *Candida albicans*

**DOI:** 10.1371/journal.ppat.1004365

**Published:** 2014-09-18

**Authors:** Linda M. Holland, Markus S. Schröder, Siobhán A. Turner, Heather Taff, David Andes, Zsuzsanna Grózer, Attila Gácser, Lauren Ames, Ken Haynes, Desmond G. Higgins, Geraldine Butler

**Affiliations:** 1 School of Biomedical and Biomolecular Science, Conway Institute, University College Dublin, Belfield, Dublin, Ireland; 2 Departments of Medicine and Microbiology and Immunology, University of Wisconsin, Madison, Madison, Wisconsin, United States of America; 3 Department of Microbiology, University of Szeged, Szeged, Hungary; 4 School of Biosciences, University of Exeter, Exeter, Devon, United Kingdom; 5 School of Medicine and Medical Science, Conway Institute, University College Dublin, Belfield, Dublin, Ireland; University of Rochester, United States of America

## Abstract

*Candida parapsilosis* and *Candida albicans* are human fungal pathogens that belong to the CTG clade in the Saccharomycotina. In contrast to *C. albicans*, relatively little is known about the virulence properties of *C. parapsilosis*, a pathogen particularly associated with infections of premature neonates. We describe here the construction of *C. parapsilosis* strains carrying double allele deletions of 100 transcription factors, protein kinases and species-specific genes. Two independent deletions were constructed for each target gene. Growth in >40 conditions was tested, including carbon source, temperature, and the presence of antifungal drugs. The phenotypes were compared to *C. albicans* strains with deletions of orthologous transcription factors. We found that many phenotypes are shared between the two species, such as the role of Upc2 as a regulator of azole resistance, and of *CAP1* in the oxidative stress response. Others are unique to one species. For example, Cph2 plays a role in the hypoxic response in *C. parapsilosis* but not in *C. albicans*. We found extensive divergence between the biofilm regulators of the two species. We identified seven transcription factors and one protein kinase that are required for biofilm development in *C. parapsilosis*. Only three (Efg1, Bcr1 and Ace2) have similar effects on *C. albicans* biofilms, whereas Cph2, Czf1, Gzf3 and Ume6 have major roles in *C. parapsilosis* only. Two transcription factors (Brg1 and Tec1) with well-characterized roles in biofilm formation in *C. albicans* do not have the same function in *C. parapsilosis*. We also compared the transcription profile of *C. parapsilosis* and *C. albicans* biofilms. Our analysis suggests the processes shared between the two species are predominantly metabolic, and that Cph2 and Bcr1 are major biofilm regulators in *C. parapsilosis*.

## Author Summary

*Candida* species are among the most common causes of fungal infection worldwide. Infections can be both community-based and hospital-acquired, and are particularly associated with immunocompromised individuals. *Candida albicans* is the most commonly isolated species and is the best studied. However, other species are becoming of increasing concern. *Candida parapsilosis* causes outbreaks of infection in neonatal wards, and is one of the few *Candida* species that is transferred from the hands of healthcare workers. *C. parapsilosis*, like *C. albicans*, grows as biofilms (cell communities) on the surfaces of indwelling medical devices like feeding tubes. We describe here the construction of a set of tools that allow us to characterize the virulence properties of *C. parapsilosis*, and in particular its ability to grow as biofilms. We find that some of the regulatory mechanisms are shared with *C. albicans*, but others are unique to each species. Our tools, based on selectively deleting regulatory genes, will provide a major resource to the fungal research community.

## Introduction

More than 300 *Candida* species have been described to date [1]. Although all *Candida* species are Ascomycetes (belonging to the Saccharomycetales), they are paraphyletic, and do not share a recent common ancestor [2]. As a result, they have few shared characteristics. The term “*Candida*” suggests that they are asexual species, but sexual or cryptic sexual cycles are increasingly being identified [3], [4], [5], [6]. Most well studied *Candida* species belong to the monophyletic CTG clade, where the codon CTG is translated as serine rather than leucine [2], [7]. These include the major human fungal pathogens *Candida albicans*, *Candida parapsilosis* and *Candida tropicalis* [8], [9]. Whereas *C. albicans* is still the most common cause of candidiasis, *C. parapsilosis* and the non-CTG clade species *Candida glabrata* are increasing in frequency [8], [9], [10]. Properties of *C. albicans* associated with the ability to cause disease have been well characterized, and include growth in yeast and hyphal forms, epigenetic switching from white to opaque cells, secretion of hydrolases, and adhesion and biofilm development (reviewed in [11]). While some of these properties are likely to be shared with other CTG-clade species, many are species or lineage specific. For example, only *C. albicans* and its close relative *Candida dubliniensis* can grow in truly hyphal forms, and white-opaque switching and the associated parasexual cycle have only been described in *C. albicans*, *C. dubliniensis* and *C. tropicalis* [12], [13], [14]. *C. parapsilosis*, a major cause of infection in premature neonates [15], does not appear to have a sexual cycle [16], [17] and does not undergo white-opaque switching [18]. Unlike other *Candida* species, *C. parapsilosis* is often isolated from the hands of health care workers and has been responsible for causing outbreaks of infection [19], [20], [21], [22], [23], [24]. *C. parapsilosis* is responsible for approximately 20% of *Candida* infections particularly in infants less than 1 year old [25], [26].

One of the major factors of *Candida* species associated with pathogenicity is their ability to grow as biofilms on implanted medical devices [27]. Biofilms are composed of communities of microorganisms associated with a surface and embedded in an extracellular matrix, and are believed to be the major growth form of microorganisms in nature [28]. Biofilms are extremely refractory to antimicrobial therapy and treatment usually involves removal of the infected device. Biofilm formation in *C. albicans* has been well characterized and occurs in several stages (reviewed in [29], [30]). The first step involves yeast cells adhering to a substrate surface. This is followed by a period of cellular growth, or biofilm initiation. During the maturation stage, hyphae are produced and cells become encased in an extracellular matrix (ECM). The final stage is dispersal, when yeast cells break away from the biofilm structure and disseminate around the body to seed new sites of infection [31], [32].

Although many *Candida* species form biofilms, the structures are highly variable [28], [33]. *C. albicans* biofilms consist of a compact basal layer of yeast cells and a thicker less compact hyphal layer all surrounded by an ECM composed mainly of carbohydrate [34]. In contrast *C. parapsilosis* does not make true hyphae, and biofilms are composed of yeast and pseudohyphal cells only [27], [35], [36]. The ability of *C. parapsilosis* to produce biofilm is also highly strain dependent [28], [33].

Many of the key regulators of biofilm formation in *C. albicans* have been identified (reviewed in [37]). Hyphal formation is a pivotal step, and mutants blocked in filamentation are often impaired in biofilm development [38]. Nobile et al [39] identified a network of six transcription factors (*BCR1*, *EFG1*, *TEC1*, *ROB1*, *NDT80* and *BRG1*) that play a major role in regulating *C. albicans* biofilm growth. In addition, Finkel et al [40] identified 30 transcription factors required for adhesion, some of which (such as Bcr1) are also necessary for mature biofilm development. We have previously shown that *C. parapsilosis* orthologs of *BCR1* and *EFG1* are required for biofilm formation in this species [18], [35]. However, even though the function of the transcription factors is at least partially conserved, many of the gene targets are different, and some conserved targets of Bcr1 have different functions [41]. For example the CFEM family of cell wall proteins is required for biofilm development in *C. albicans*, but not in *C. parapsilosis* [41].

We report here the construction of the first large-scale gene deletion collection in *C. parapsilosis*, targeting 100 genes representing transcription factors, protein kinases and species-specific genes. We carry out a detailed comparison of *C. parapsilosis* and *C. albicans* phenotypes, particularly in relation to biofilm development. We find that overall, the molecular function of orthologous genes is generally conserved between the two species. However, there are also important differences. *BRG1* and *TEC1*, transcription factors required for biofilm development in *C. albicans*, do not have the same role in *C. parapsilosis*. *CZF1*, *UME6*, *CPH2* and *GZF3* are regulators of biofilm development in *C. parapsilosis* only.

## Results

### Construction of a gene knockout collection in *Candida parapsilosis*

**Figure 1:**
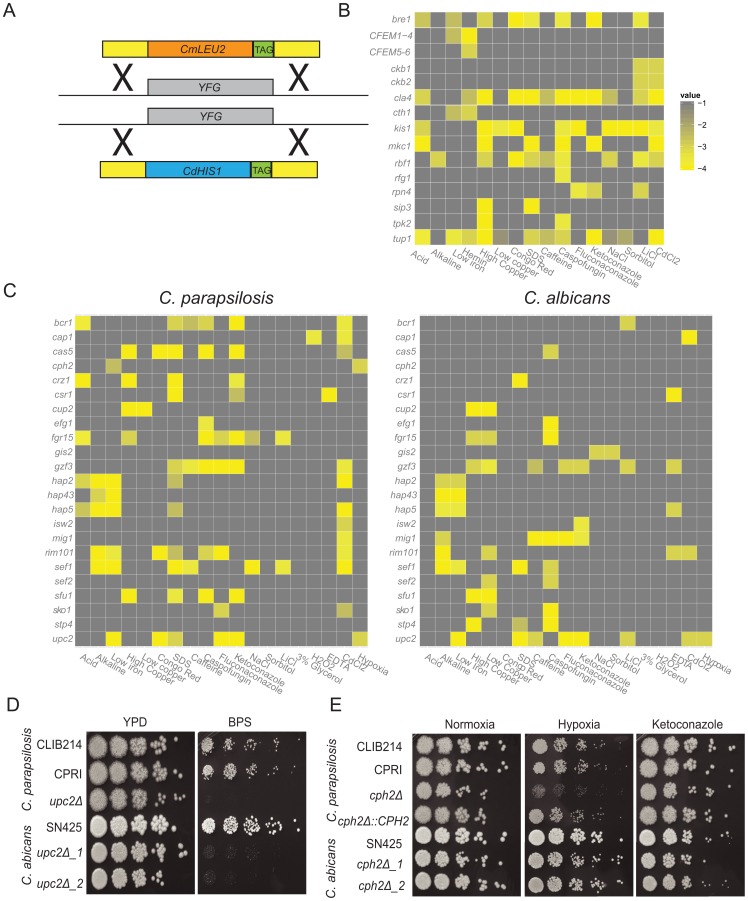
Phenotype screening of *C. parapsilosis* gene knockout strains. (A) Both alleles of 100 *C. parapsilosis* genes (YFG) were deleted by replacement with *C. maltosa LEU2* and *C. dubliniensis HIS1* by homologous recombination. Unique barcode sequences (TAG) were included in each deletion. Two independent replicates of each strain were constructed. (B,C) Each deletion strain was grown overnight in a 96 well plate in YPD media, diluted 1∶100 into fresh YPD and replica plated to test media using a 48-pin replicator. Each plate contained the control strains CLIB214 and CPRI in duplicate. Growth of each deletion strain was scored in comparison to the control strains. Strains that were scored in *C. parapsilosis* only are shown in (B), and strains with a detectable phenotype in both *C. albicans* and *C. parapsilosis* are shown in (C). Only scores <−2.5 are shown, and isolates with no growth defect on the selected media are not included. The full data set is available in Table S2. (D) Deleting *UPC2* in both *C. parapsilosis* and *C. albicans* results in reduced growth in low iron conditions (YPD, and YPD+0.15 mM BPS). (E) Cells were grown overnight in YPD, diluted in PBS and plated on YPD containing 0.005 µg ml^−1^ ketoconazole where indicated, and incubated at 30°C in 21% or 1% O_2_ for 2 days. Deleting *CPH2* in *C. parapsilosis* results in a growth defect under hypoxic (1% oxygen) conditions, but there is no effect on the equivalent deletion of *C. albicans*. Growth is restored by re-introducing one allele of *CpCPH2*. There is no effect on ketoconazole sensitivity in either species.

Most of the available gene disruption collections in *C. albicans* target transcription factors or protein kinases [42], [43], [44], [45]. We therefore selected similar genes from *C. parapsilosis* (Figure 1, Tables S1, S2). In total, we chose genes encoding 73 transcription factors, 16 protein kinases, 1 putative RNA-binding protein, 1 putative tRNA-methyl transferase, 6 genes that are apparently unique to *C. parapsilosis*, and members of the CFEM family of transmembrane proteins [41], [46], [47]. We selectively deleted entire open reading frames rather than generating random insertions, to facilitate downstream analysis. The *C. parapsilosis* genome is diploid and therefore requires two rounds of gene disruption to create a homozygous mutant. Gene disruptions in *C. parapsilosis* have previously been carried out using a recyclable *SAT1* (nourseothricin resistance) cassette [35], [36]. Although this approach is successful, it is slow and very inefficient. Instead, we adapted a fusion PCR method previously developed for gene deletion in *C. albicans*, in which each allele is replaced with a heterologous selectable marker [45] (Figure 1A, Figure S1).

Deletions were constructed in the *C. parapsilosis* type strain, CLIB214. Auxotrophic mutations in *LEU2* and *HIS1* were first generated using a *SAT1* cassette, yielding strain CPL2H1 (see methods) (Figure S1, Table S1) [35]. Candidate genes were then deleted by replacing one allele with *HIS1* from *C. dubliniensis*, and one with *LEU2* from *C. maltosa* (Figure 1A, Figure S1). All mutant strains were confirmed by PCR using primers inside *CdHIS1* or *CmLEU2* and a primer outside of the integration sites at both the 5′ and 3′ end of the gene. Deletion of the target open reading frame was also confirmed using PCR. A control strain (CPRI) was created by integrating *CdHIS1* and *CmLEU2* at the site of the original *HIS1* alleles (Figure S1). Both CLIB214 and CPRI strains were used as controls for the majority of experiments.

Two independent homozygous deletion mutants were generated for each targeted gene, which increases the probability that an observed phenotype in both strains is a result of the gene deletion, and not from a secondary effect. A unique 20 base pair sequence tag (barcode) was also incorporated into each mutant strain, which will facilitate future competition experiments. In total, 200 barcoded deletion strains were constructed (Table S1).

### Phenotypic screen of deletion collection

Growth of the deletion collection was determined in 42 different conditions, designed to identify nutritional, cell wall, osmotic and oxidative stress phenotypes, and response to antifungal drugs (Table S2). Some strains had severe growth defects, and were not included in the phenotype analysis (Table S2). For the remainder, each independent strain was grown in YPD in 96 well plates (two replicates per candidate gene), and then replica-plated using a 48-pin replicator to selective media. YPD was used as the base media except where the effect of different carbon sources was tested. Several drugs and chemicals were tested at a range of concentrations (Table S2). Growth was scored using a simple scoring system (−4 to +1, where 0 is the same as the control strains). The scores were averaged between sister strains and replicate screens and were converted to heatmaps (Figure 1B,C). Of the 73 transcription factor deletions constructed in *C. parapsilosis*, 64 orthologous deletions were available from a large scale screen in *C. albicans* [43], and another (*UME6*) was obtained from Banerjee et al [48]. Growth of the *C. albicans* strains was monitored under similar conditions as for *C. parapsilosis* except that lower concentrations of ketoconazole, fluconazole and caspofungin and higher concentrations of CdCl_2_ were used, and utilization of heme was not tested (Table S2). Nine transcription factor deletions are available in *C. parapsilosis* only. The phenotypes of these, and of the *C. parapsilosis* protein kinase deletions, are described in the supporting information (Text S1, Table S2).

Figure 1C shows a comparison of the phenotypic profiles of orthologous deletions in the two species. Thirty-five gene deletions are not shown because they have no, or little, effect on growth in any condition tested for either species (Table S2). Changes in colony morphology were not recorded. Another four strains (deletions of *ACE2*, *NRG1*, *SSN6* and *TUP1*) were removed because the phenotypes are difficult to score in *C. albicans* or in both species, mostly because they significantly affect filamentation. Deleting *RBF1* and *RPN4* in *C. albicans* and *NDT80* in *C. parapsilosis* results in dramatic reduction in growth; the *NDT80* deletion is therefore not shown, and the *RBF1* and *RPN4* deletions are included with the *C. parapsilosis*-only data (Figure 1B). Many phenotypes previously described in *C. albicans* are shared in *C. parapsilosis*. These include the role of *CAP1* in the oxidative stress response (sensitivity to cadmium chloride [49]), enhanced sensitivity of *CSR1* deletion to metal chelators (EDTA; [43]) and the role of *HAP2*, *HAP5*, *HAP43* and *SEF1* in regulating the response to iron (deletions have reduced growth to low iron, resulting from addition of the iron chelator BPS [50], [51]), of *RIM101* as a regulator of the response to alkaline conditions [42], and *UPC2*, which determines sensitivity to azole drugs [52], [53]. The function of many of these regulators is conserved across a wide evolutionary distance, at least since the common ancestor with *S. cerevisiae* [54], [55], [56], [57], [58], [59], [60]. Regulation of the iron and copper response is similar in both species though there are some subtle differences (Text S1).

Some phenotypes conserved between the two species have not previously been reported. These include the sensitivity of the *upc2* deletions to the presence of the iron chelator, BPS (Figure 1D). Upc2 is also required for growth on xylose as the main carbon source in both *C. albicans* and *C. parapsilosis* (Table S2). However, despite the overall similarity, there are also significant differences between the two species. Several deletion strains (e.g. *BCR1, CPH2, GIS2, ISW2, MIG1, SEF2, STP4*) have no shared phenotypes. Some gene deletions have pleiotropic effects in *C. parapsilosis*; for example, deleting *SEF1* results in reduced growth in many conditions, whereas the equivalent deletion in *C. albicans* results in much fewer phenotypes (Table S2). One of the most obvious differences between *C. albicans* and *C. parapsilosis* occurs during growth in low oxygen (hypoxic) conditions (Table S2, Figure 1C,E). Deleting *UPC2* confers sensitivity to hypoxia in both, as previously reported [43], [53], [61], [62]. No other *C. albicans* gene deletion tested affects hypoxic growth. However, in *C. parapsilosis*, deleting *CPH2* also reduces growth in hypoxia, and growth is restored when the wildtype gene is reintroduced (Figure 1E). In addition, we have previously shown that expression of *CpCPH2* is increased during growth in hypoxia [63]. However, deleting *CpCPH2* does not affect sensitivity to azole drugs (Figure 1E).

We have previously shown that in *C. parapsilosis*, similar to *C. albicans*, members of the CFEM family are important for heme utilization [41], [64], [65]. Whereas *C. albicans* has five family members, in *C. parapsilosis* the family has expanded to seven (*CFEM1-7*). Here, we deleted *CFEM1-CFEM4* together, *CFEM5* and *CFEM6* together, and *CFEM7* alone (Table S2). Similar to previous reports, strains missing *CFEM1*-*CFEM4* or *CFEM5*-*CFEM6* are unable to use heme as a sole source of iron (Figure 1B, Table S2, [41]). However, deleting *CFEM7* alone had no effect (Table S2). We also find that deleting *CTH1* (CPAR2_407950) renders cells sensitive to iron, and unable to use hemin as a sole source of iron (Figure 1B). Expression of *CpCTH1* is induced in low iron conditions [41]. *CpCTH1* is an ortholog of both members of the *CTH1/CTH2* gene pair in *S. cerevisiae*, proteins that bind to RNA transcripts from iron metabolic genes, targeting them for degradation [66], [67]. Our results suggest that *CTH1* forms part of the iron regulatory pathway in *C. parapsilosis*.

### Identification of regulators of biofilm development

**Figure 2:**
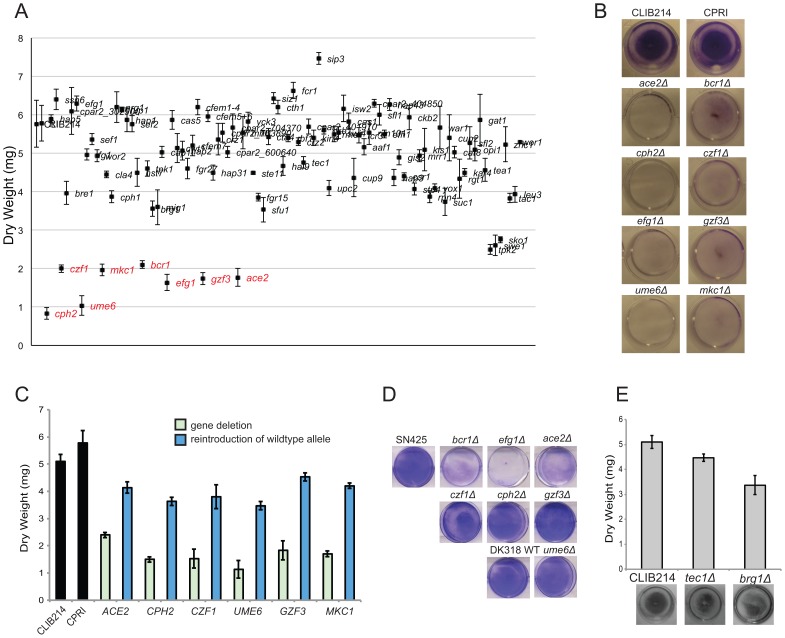
Identification of biofilm regulators in *C. parapsilosis*. (A) 95 *C. parapsilosis* knockout strains (Table S2) and two control strains were grown under standard biofilm conditions in 6 well plates. Two wells were combined for each strain, and the dry weight of the biofilm was determined. The standard deviation from three independent measurements is shown. Strains with statistically significant reductions in dry weight are indicated in red (p<0.0005). (B) The indicated *C. parapsilosis* strains were grown under standard biofilm conditions in 24 well plates, and the biofilm was stained with crystal violet. (C) A wild type copy of each gene was re-introduced into the relevant *C. parapsilosis* mutant strain as indicated, and the biofilm biomass was measured. Re-introduction of one allele restored biofilm to levels similar to the control strains, CLIB214 and CPRI. (D) *C. albicans* strains with the indicated gene deletions were grown in 6 well plates in Spider media for 48 h, and stained with crystal violet. The relevant *C. albicans* wild type strains were included for comparison. Deleting *BCR1*, *EFG1* and *ACE2* dramatically reduces biofilm formation, but deleting *CPH2*, *CZF1*, *GZF3*, or *UME6* has little effect. (E) Biofilms from *C. parapsilosis* strains carrying deletions of *TEC1* and *BRG1* were grown in 24 well plates in SD media with 50 mM glucose and were stained with crystal violet (bottom) or dry weights were measured (top). The gene deletions have no significant effect on biofilm formation.

The effect of the *C. parapsilosis* gene deletions on biofilm development on polystyrene surfaces was determined visually using crystal violet staining, and by measurement of biomass (dry weight). Several deletion strains (*ADA2*, *MSS2*, *VPS34*, *NDT80* and *YCK2*) exhibited growth defects on YPD and were not included in the biofilm screen. Of the 95 unique deletions tested, eight had obvious visual defects and significantly reduced dry weight formation, including seven transcription factors (*EFG1*, *CZF1*, *GZF3*, *UME6*, *CPH2*, *BCR1* and *ACE2*) and one protein kinase (*MKC1*) (Figure 2A,B). Some other deletion strains had minor effects on biofilm growth, but only these eight had significant and reproducible reductions in biofilm mass. Reintroducing the intact genes restored biofilm growth (Figure 2C; restoring *BCR1* and *EFG1* have been described previously [18], [35]). These mutants displayed no significant defects in growth in liquid culture in biofilm media, except that the *ACE2* deletion has a cell-separation defect [68].

A recent study identified a network of six transcription factors (*BCR1*, *EFG1*, *TEC1*, *NDT80*, *BRG1* and *ROB1*) that regulate biofilm formation in *C. albicans* [39]. In a separate study, *ACE2* was shown to be required for adhesion and subsequent biofilm development in the same species [40]. To determine the overlap between the networks regulating biofilm growth in *C. albicans* and *C. parapsilosis*, we directly compared the effect of deleting the orthologous transcription factors in the two species (Figure 2D,E). Both species were grown in conditions that maximize biofilm development [35], [38], [41]. We confirmed that deleting *BCR1*, *EFG1* and *ACE2* reduces biofilm development in *C. albicans*, similar to *C. parapsilosis* (Figure 2D), whereas deleting *CZF1*, *GZF3*, *UME6*, or *CPH2* has little effect on *C. albicans* biofilm growth in our assay (Figure 2D).

From the remaining genes in the *C. albicans* biofilm regulatory network, orthologs of *TEC1*, *NDT80*, and *BRG1* were not identified in our large-scale screen of biofilm-defective mutants in *C. parapsilosis* (Figure 2A). We confirmed that deleting *TEC1* or *BRG1* did not dramatically reduce *C. parapsilosis* biofilm formation in follow-up tests (Figure 2E). However, we could not determine the role of the *NDT80* ortholog, because unlike in *C. albicans*, deleting *NDT80* in *C. parapsilosis* results in a significant growth defect. There is no ortholog of the final member of the *C. albicans* network, *ROB1*, in the *C. parapsilosis* genome [39], [47].

### Structure of *C. parapsilosis* biofilms

**Figure 3:**
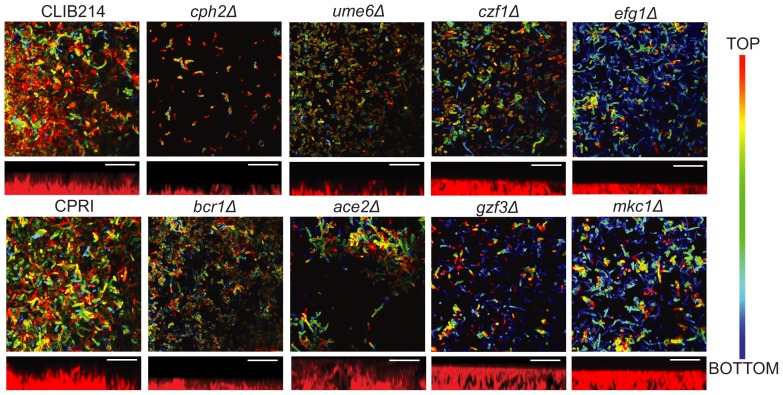
Deleting regulators alters biofilm structure of *C. parapsilosis*. Biofilm structure was determined using confocal microscopy. Biofilms from the indicated strains were grown on Thermanox slides in SD media with 50 mM glucose, stained with conA and visualized using a Zeiss LSM510 confocal scanning microscope. False-color images are shown. Side view images showing the approximate thickness of the biofilm were created using Fiji. Images were restricted on the vertical scale to prevent aberrant measurements from small regions of the biofilm. Scale bars (20 µm) are shown. CLIB214 (11 µm), CPRI (15 µm), *cph2Δ* (4 µm), *ume6Δ* (6 µm), *czf1Δ* (10 µm), *efg1Δ* (6 µm), *bcr1Δ* (5 µm), *ace2Δ* (20 µm), gzf3Δ (12 µm), *and mkc1Δ* (11 µm).

We used confocal laser scanning microscopy (CLSM) to visualize the morphology and structure of *C. parapsilosis* biofilms growing on the surface of Thermanox slides and stained with concanavalin A [63] (Figure 3). The control strains produce a compact biofilm consisting of layers of yeast and pseudohyphal cells. The biofilm layer is thinner than previously reported (10–20 µm, [35]), but it is reproducible.

Deleting *CPH2*, *UME6*, *EFG1* and *BCR1* in *C. parapsilosis* results in biofilms with very few layers that are consistently thinner than those produced by the control strains. Biofilms generated by *CPH2*, *UME6* and *CZF1* deletions are mainly composed of yeast cells, with few pseudohyphal cells present, whereas the *BCR1* mutant produces a thin biofilm of both yeast and pseudohyphal cells. The *ACE2* deletion generates patchy biofilm, consisting of clumps of pseudohyphal cells across the surface of the slide, probably due to the cell separation defect caused by this mutation [68]. Biofilms produced by the *GZF3* and *MKC1* deletion strains have both yeast and pseudohyphal cells present in several layers; however the biofilm produced is not as compact as the wild type (Figure 3). We have previously shown that deleting *EFG1* increases morphological switching between “wrinkled” and “smooth” colonies, both of which have reduced biofilm development [18]. We did not differentiate between different colony morphologies in the assay presented here. However, *CPH2*, *UME6* and *CZF1* deletion strains all have reduced colony wrinkling compared to wildtype (not shown).

### Characterization of biofilm defective mutants *in vivo*

**Figure 4:**
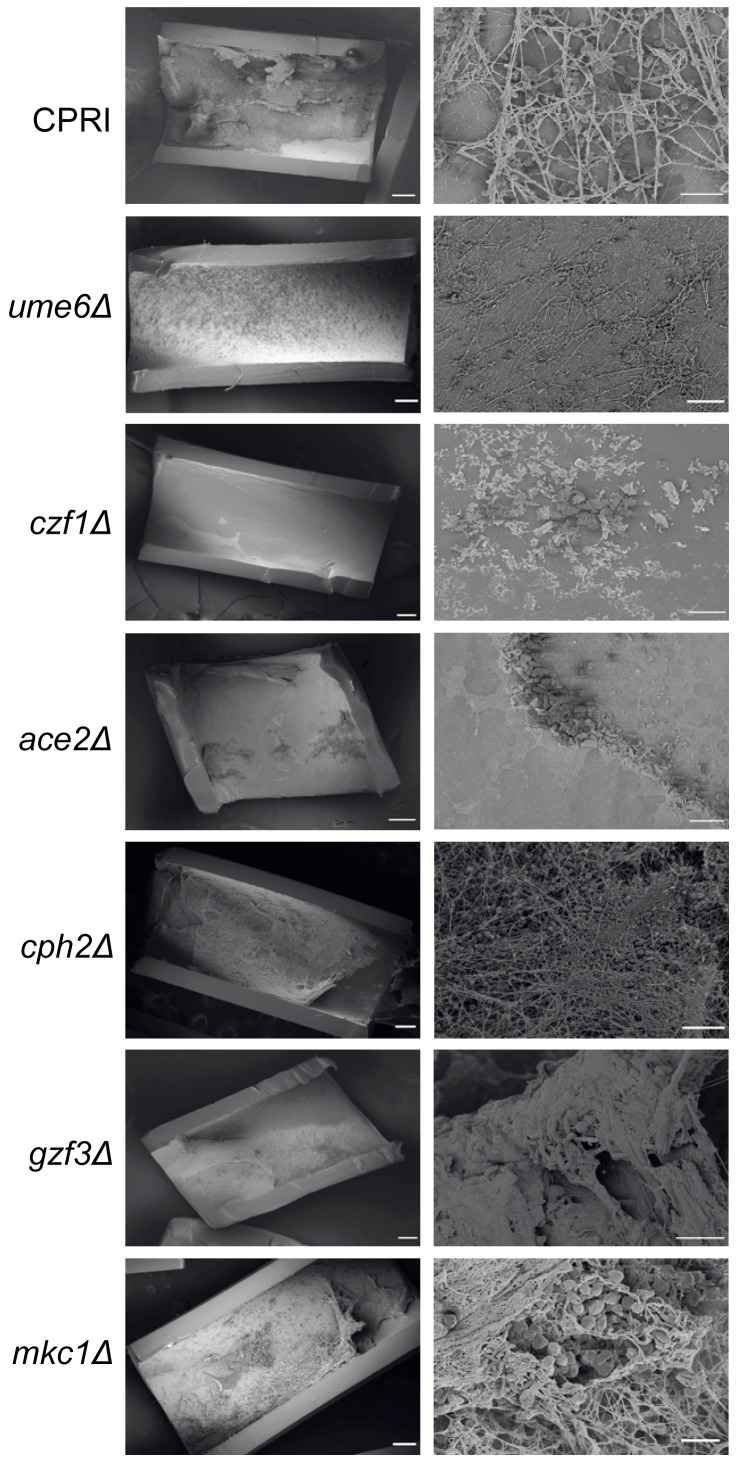
Deletion of regulators disrupts biofilm formation *in vivo*. The indicated *C. parapsilosis* strains were inoculated into catheters in the rat CVC model, and biofilms were visualized by scanning electron microscopy (SEM) after 24 hours. Deleting *UME6*, *CZF1* and *ACE2* reduces biofilm formation in comparison to the control strain, CPRI, whereas deleting *CPH2*, *GZF3* and *MKC1* has little effect. *BCR1* and *EFG1* deletions were assayed in this model previously [18], [41]. Scale bars correspond to 200 µm and 20 µm.

Biofilm development *in vivo* is substantially different from the *in vitro* models used here. In particular, biofilms formed in catheters undergo stress from blood flow. We therefore used an established rat central venous catheter (CVC) model of infection [69] to test the effect of deleting regulatory genes in *C. parapsilosis* (Figure 4). We compared the deletion constructs to the control CPRI strain, because biofilm development by the wildtype strain was variable in this model. *C. parapsilosis* biofilms *in vivo* consist of yeast cells, matrix and host cells. We have previously shown that deleting *BCR1* greatly reduces biofilm formation *in vivo*, whereas deleting *EFG1* has a more minor effect [18], [41]. Here we show that deleting *UME6* and *CZF1* also greatly reduce biofilm formation (Figure 4). The biofilm from the *UME6* deletion consists of a single layer of yeast cells with little obvious matrix, whereas the *CZF1* deletion produces little obvious biofilm. Deleting *ACE2* results in a clumpy biofilm. In contrast to the *in vitro* assay, deleting *CPH2*, *GZF3* and *MKC1* has little effect on biofilm growth *in vivo*.

### Transcriptional profile of *C. parapsilosis* biofilms

We previously used microarray analysis designed from a partial genome sequence to determine the transcriptional profile of *C. parapsilosis* biofilms growing under flow conditions in a fermenter [63]. In general, we found that the transcriptional response of biofilms is similar to that of cells growing in hypoxic conditions, and is associated with upregulation of fatty acid metabolism genes. Here, we used RNA-seq analysis to characterize the transcription profile of cells growing in static conditions, the same as those used to identify biofilm regulators. Compared to planktonic conditions, 777 genes are up-regulated and 662 genes are down-regulated in biofilms (log_2_FC+/− 1.5, adjusted p-value <0.001) (Table S3). Upregulated genes are enriched for processes associated with lipid/fatty acid oxidation and transmembrane transport, whereas downregulated genes are enriched in translation, macromolecule and amino acid biosynthetic processes and cellular component assembly (Table S4).

**Figure 5:**
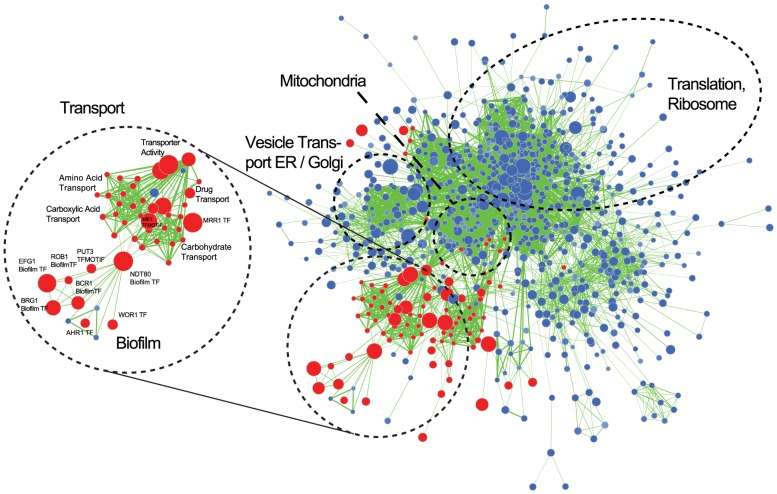
Gene Set Enrichment Analysis of the *C. parapsilosis* biofilm transcriptome. Visualization of gene sets enriched in the *C. parapsilosis* wild type biofilm transcriptome compared to *C. parapsilosis* planktonic transcriptome. Gene sets are obtained from *C. albicans* and *S. cerevisiae* data [70]. *C. albicans* gene sets enriched in genes up-regulated in *C. parapsilosis* biofilms are shown in red, and gene sets enriched in down-regulated genes are in blue. The size of the nodes represents the number of genes in the gene sets in *C. albicans*, and the edges represent an overlap of genes between gene sets. Sub-networks of interest are highlighted in black circles. “TF” indicates dataset from *C. albicans* transcription factor deletions, and “Biofilm” indicates the *C. albicans* biofilm regulatory network form Nobile at al [39]. A larger version of the figure is available as Figure S2, and the Cytoscape data is provided as Dataset S1.

We used Gene Set Enrichment Analysis (GSEA) to identify gene categories that are over-represented in the biofilm transcriptome. GSEA allows the identification of statistically significant overlaps between a ranked gene list (e.g. the *C. parapsilosis* biofilm transcriptome), and gene lists (or gene sets) identified in other experimental analyses. We used a collection of 8,852 gene sets derived from microarray and ChIP (chromatin immunoprecitation) experiments from *C. albicans* and from protein-protein interactions from *S. cerevisiae* that were collected and described by Sellam et al [70]. We extracted a set of *C. albicans* orthologs of our ranked list of genes differentially expressed in *C. parapsilosis* biofilms, and looked for similarities between this gene list and the gene sets described by Sellam et al [70]. The network of similar gene sets was visualized using Cytoscape [71] (Figure 5, Figure S2). In these figures, nodes represent gene sets, and edges connect nodes sharing a significant number of genes. Clustering algorithms in Cytoscape group highly interconnected and similar gene sets together. We have colored nodes that included genes upregulated in *C. parapsilosis* biofilms in red, and those that include downregulated genes in blue. Figure 5 shows that upregulated genes share similarities with gene sets associated with transport (including carboxylic acid and drug transport) in *C. albicans*. There is also a significant overlap with the *C. albicans* biofilm regulatory network described by Nobile et al [39]. The transport and biofilm networks are connected via Ndt80 (Figure 5). Downregulated genes are enriched for processes associated with translation and the ribosome.

### Comparison to *C. albicans* biofilm transcriptome

**Figure 6:**
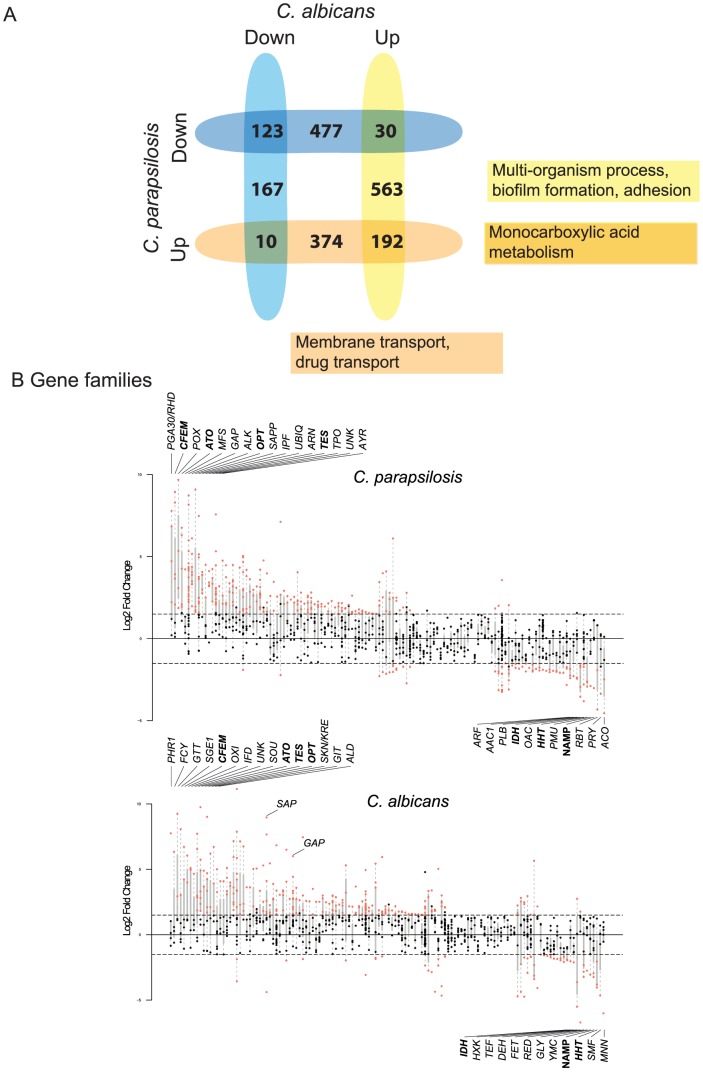
Transcriptional profiling of *C. parapsilosis* biofilms. Comparison of genes differentially expressed in *C. parapsilosis* biofilms with *C. albicans* biofilms (from [39]). The intersections show genes that are differentially regulated in biofilms in both species, and the remaining numbers indicate genes that differentially regulated in one species only. Up (blue) and down (yellow) regulated genes are shown separately. The most common GO processes in upregulated genes are indicated. Gene families enriched in differentially expressed genes in biofilms in *C. parapsilosis* (top) and *C. albicans* (bottom). Each vertical line represents a gene family. Families are ordered with respect to mean expression from highest (left) to lowest (right). Each dot represents an individual gene; those that are differentially expressed are highlighted in red. The families with the highest and lowest expression are named, as are others discussed in the text. Families highlighted in bold text are highly differentially expressed in biofilms in both species.

The similarity between the *C. parapsilosis* biofilm transcriptome and the *C. albicans* biofilm regulatory network prompted us to directly compare the biofilm transcriptional profiles of the two species, using the *C. albicans* data from Nobile et al [39] (Figure 6A). In *C. albicans* 785 genes are up-regulated and 300 genes down-regulated in biofilm compared to planktonic cells. There are 192 genes upregulated in biofilms in both species (Figure 6A). These are enriched in oxidoreductases and in pathways associated with carboxylic acid metabolism. Interestingly, genes upregulated in *C. albicans* only (and not in *C. parapsilosis*) are enriched in processes associated with biofilm formation and adhesion. Many are transcription factors, some of which we have shown have no role in biofilm development in *C. parapsilosis* (*BRG1*, *WAR1* [40], *CRZ2* [40] and *ZNC1* [40]), and others that we have not tested, but which are not differentially expressed in biofilms e.g. *GCN4* [72]. Genes upregulated only in *C. parapsilosis* are enriched in pathways associated with transmembrane and drug transport. This category includes a large number of genes with no annotation, suggesting that their function may be specific to *C. parapsilosis*.

One of the difficulties comparing gene expression profiles between species is the reliance on the identification of orthologs present in both. This may underestimate the importance of genes that are found in one species only. In addition, it is very difficult to evaluate the roles of gene families that have different numbers or members in different *Candida* species [73]. We therefore categorized the gene families in *C. albicans* and *C. parapsilosis* using Markov Clustering (MCL; [74]) and looked for evidence of family enrichment among differentially expressed genes in biofilms (again using the data from [39] for *C. albicans*). Figure 6B shows the gene families ordered by average gene expression for both species (from highest to lowest expression; full data in Table S5).

The CFEM family, the ATO family of putative ammonia transporters [75], the OPT family of oligopeptide transporters [76] and the TES family of acyl CoA-thioesterases are among the most enriched among upregulated genes in both species (Figure 6, Table S5). General amino acid permeases (GAP family) and secreted aspartyl proteases (SAP family) are also among the most highly expressed in *C. parapsilosis*, and are enriched among up-regulated genes in *C. albicans* (Figure 6B). The most downregulated genes enriched in both species include a family involved in Nucleic Acid Metabolic Processes (NAMP) (including *TUP1*, a major hyphal regulator in *C. albicans* that is required for biofilm growth [77]) and histone genes (HHT family). The *PGA30/RHD3* family has the highest average expression in *C. parapsilosis*; these encode GPI-anchored proteins predicted to be localized to the cell wall [78].

### Identification of targets of biofilm regulators

**Figure 7:**
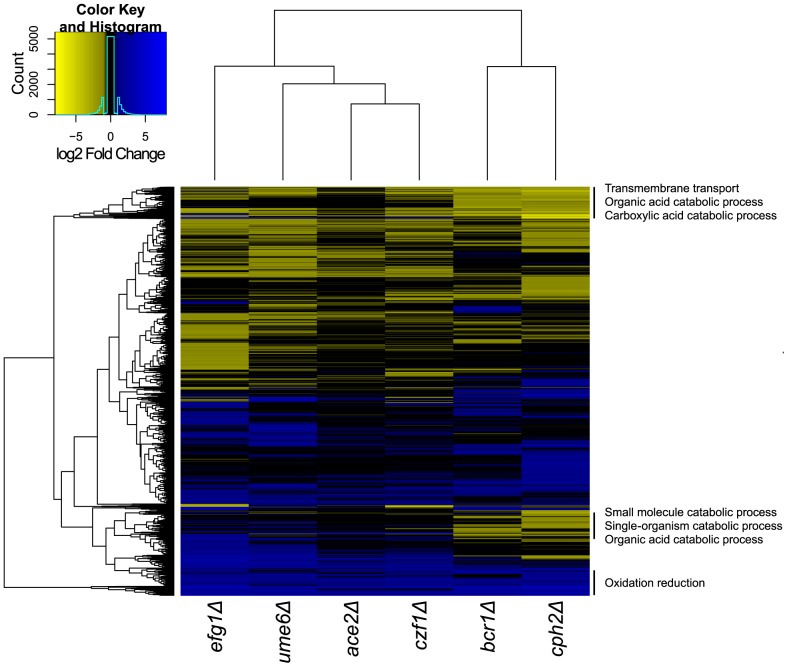
Comparison of transcription profiles of biofilm regulators. Gene expression profiles from the indicated *C. parapsilosis* strains were determined using RNA-seq of three independent replicates and compared to the profile of wildtype biofilms. Similar expression patterns were clustered using Bioconductor [114]. The major GO patterns associated with specific clusters are shown.

To identify the genes that are regulated in *C. parapsilosis* biofilms, we used RNA-seq to compare the transcriptional profiles of biofilms from strains deleted for *EFG1*, *CZF1*, *UME6*, *CPH2*, *BCR1* and *ACE2* to biofilms from wildtype strains (Figure 7). We were unable to isolate RNA of sufficient quality from strains deleted for *GZF3* or *MKC1*, suggesting that there may be changes in the extracellular matrix or cell wall that we cannot directly observe. Only seven genes are downregulated in biofilms of all deletion strains and upregulated in the wildtype (*ARO10*, *ATO1*, *PUT4*, *STE18*, *HGT17*, *CPAR2_803700*, *CPAR2_805760*). We have not yet characterized the roles of these genes in biofilm development.

**Figure 8:**
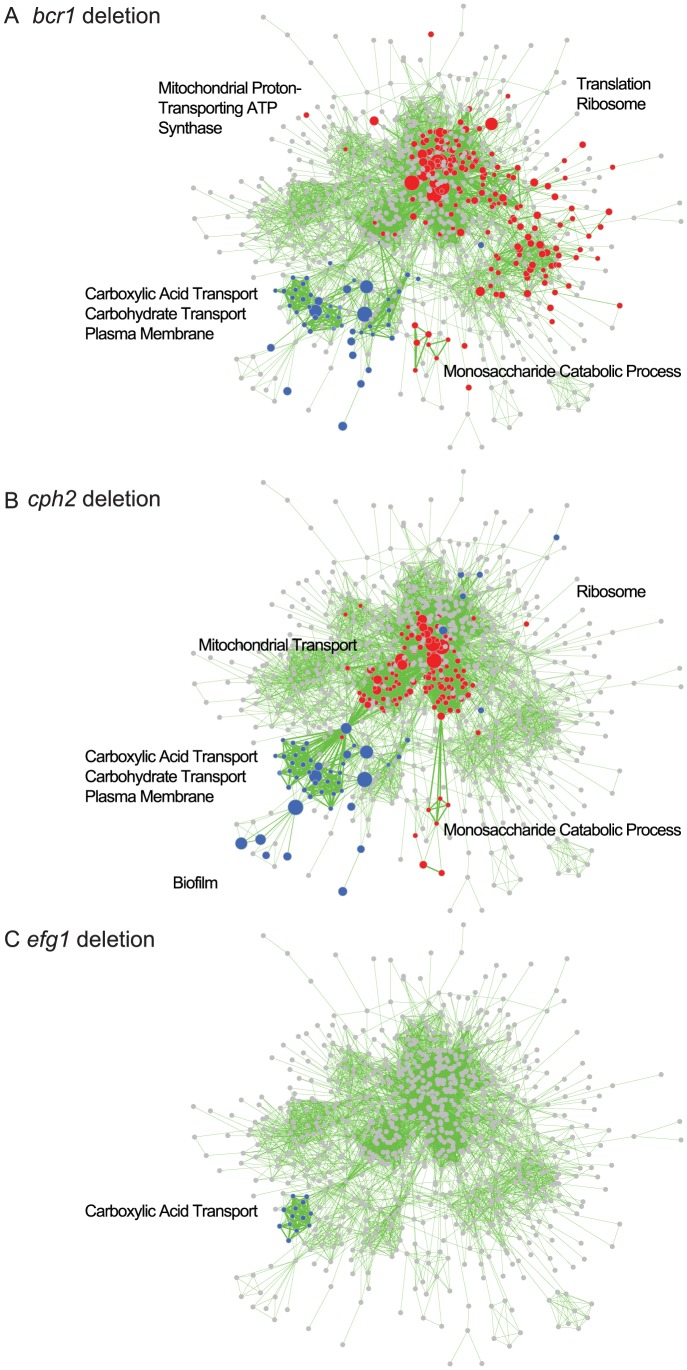
GSEA analysis of biofilms formed by *C. parapsilosis* deletion strains. The panels show enriched genesets from GSEAPreRanked analysis of biofilms from (A) *bcr1*, (B) *cph2* and (C) *efg1* deletion strains overlayed on the transcriptional profile of *C. parapsilosis* biofilms compared to planktonic cells from Figure 5. Gene sets enriched in upregulated genes in the deletion biofilms compared to wildtype biofilms are shown in red, and gene sets enriched in downregulated genes are shown in blue. Gene sets that are not changed in the deletion strains are shown in gray. Enrichment is visualized using Cytoscape, as described in Figure 5. A larger version of the figure is available as Figure S3, and the Cytoscape data is provided as Dataset S1.

Processes associated with transmembrane transport, organic acid catabolism and carboxylic acid metabolism are upregulated in most of the deletion biofilms, compared to wildtype (Figure 7). The transcriptional profiles of biofilms from the *BCR1* and *CPH2* deletion cluster close together (Figure 7), suggesting that these transcription factors regulate a core set of genes. We also used GSEA to compare the gene sets regulated by some of the transcription factors with the biofilm network described in Figure 5. Figure 8 shows that there is a high degree of overlap between the gene sets enriched in the *BCR1* and *CPH2* deletion and in the wildtype biofilm/planktonic comparison. Gene sets associated with carboxylic acid transport and with carbohydrate transport are downregulated in both. The targets of Efg1, a known biofilm regulator in *C. parapsilosis* [18] have the smallest overlap with the biofilm network (Figure 8C). Again, the overlap includes gene sets associated with carboxylic acid transport.

## Discussion

### Conservation of phenotypes between *C. albicans* and *C. parapsilosis*

The phenotype screen revealed a high conservation in the regulation of different biological processes between *C. albicans* and *C. parapsilosis*. Overall, 15 of the 23 transcription factors in Figure 1C share at least one major phenotype in *C. albicans* and *C. parapsilosis*. Some shared phenotypes, such as role of *UPC2* in the response to low iron conditions, have not previously been highlighted. However, Homann et al [43] noticed that deleting *UPC2* in *C. albicans* resulted in the accumulation of a pink color after several days of growth on BPS, possibly signaling formation of a ferrous-BPS complex. Deleting *UPC2* also results in a growth defect of *Yarrowia lipolytica* in low iron conditions [79]. It is therefore likely that this transcription factor is involved in regulating the response to iron in several fungal species.

Despite the overall similarity observed in the phenotypic screen there are some significant differences, including the role of *CPH2* in the hypoxic response of *C. parapsilosis*. In *C. albicans*, *CPH2* is a regulator of hyphal growth with no known hypoxic role [80], [81]. It has a basic helix-loop-helix (bHLH) DNA-binding domain, and is probably a remnant of the Sterol Regulatory Binding Proteins (SREBPs) that regulate sterol synthesis and the hypoxic response in filamentous fungi and in *Schizosaccharomyces pombe*; species that have no recognizable Upc2 [79], [82], [83]. Most species in the Saccharomycotina (including *Saccharomyces* and *Candida* species) have lost domains from their SREBP homologs, and the remaining remnants have no known role in regulating sterol synthesis, or in the hypoxic response [79], [83]. *Y. lipolytica*, an outgroup of the Saccharomycotina, is a notable exception in that it retains a full length SREBP, and has gained a Upc2 ortholog [79]. However, even in this species Upc2 is the main regulator of sterol synthesis, although SREBP has retained some role in regulating the hypoxic response, particularly in the control of filamentation [79]. In *C. parapsilosis*, deleting *CPH2* does not affect sensitivity to ketoconazole (Figure 1E), indicating that its role in hypoxia is unlikely to be related to regulating expression of ergosterol genes. Elucidating the role of *CPH2* in the hypoxic response of *C. parapsilosis* will require substantial further investigation. However it appears that Cph2 and SREBPs have retained a previously unsuspected role in regulating the hypoxic response in the Saccharomycotina.

### Regulation of biofilm development

We identified seven transcription factors and one protein kinase that are important regulators of *C. parapsilosis* biofilm development *in vitro* (Figure 2). Deleting *CPH2*, *GZF3* and the protein kinase *MKC1* has no effect on biofilm development in the *in vivo* rat catheter model. This suggests that biofilm development may be context dependent. A similar phenomenon has been described in *C. albicans*; for example, *CaBRG1* is required for biofilm formation *in vitro* but not *in vivo* [39], and deleting *CaRHR2* reduces biofilm formation in the central venous catheter model but not in an oral pharyngeal model [84]. However, we note that in *C. parapsilosis* the *GZF3* and *MKC1* deletions strains have minor effects on the structure of biofilms on Thermanox slides (Figure 3), suggesting that their role as biofilm regulators may be restricted to growth in specific conditions.

**Figure 9:**
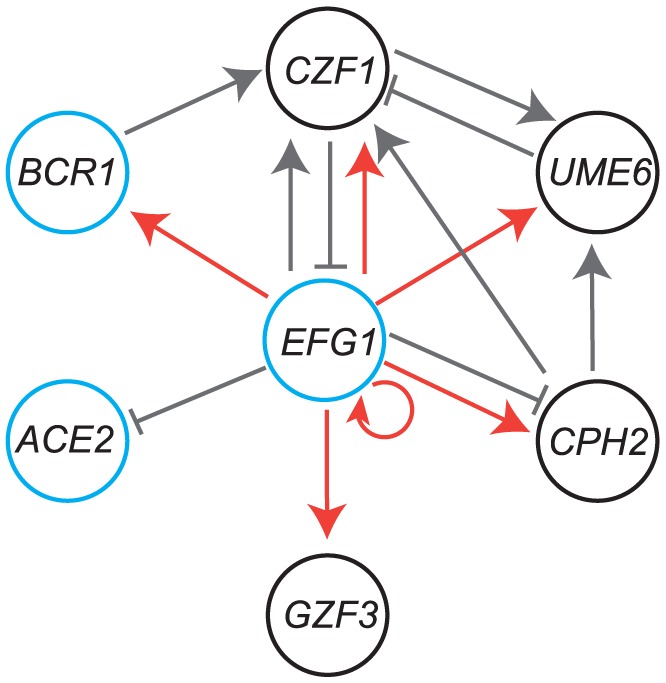
Comparison of the *C. parapsilosis* biofilm network with *C. albicans*. Genes in black circles are major regulators of biofilm development in *C. parapsilosis* only, whereas those in blue circles have a conserved role in *C. albicans*. Gray lines indicate regulation of expression from RNA-seq data; arrowheads show activation, and solid lines show repression. Red lines show direct binding of Efg1 to promoters of genes in planktonic growth, from Connolly et al [18].

Two of the *C. parapsilosis* biofilm regulators (*BCR1* and *EFG1*) are conserved with the well-characterized *C. albicans* biofilm circuit [39], and a third (*ACE2*) is also likely to regulate biofilm development in both species (Figure 9, [40], [68]). Five (*CPH2*, *UME6*, *CZF1*, *GZF3* and *MKC1*) appear to be unique to *C. parapsilosis*. We previously showed that the role of Bcr1 as a regulator of biofilm development in *C. parapsilosis* shares some similarities with that of its ortholog in *C. albicans* [85]. However, there are significant differences in the two species. For example, some of the conserved targets (e.g. the CFEM family) are required for biofilm development in *C. albicans* and not in *C. parapsilosis* [41]. The role of Bcr1 is also strain dependent. In *C. parapsilosis*, strains which make relatively low levels of biofilm (like CLIB214, the isolate used throughout this study) are Bcr1-dependent, while those that generate high levels of biofilm are not [86]. In *C. albicans*, “sexual” or “pheromone-stimulated” biofilms are made by cells that are homozygous at the mating MTL locus, and they are distinguished from the more general pathogenic biofilms. There is some evidence that both kinds of biofilms require Bcr1 [87], whereas other studies suggest that Bcr1-dependent expression of CFEM genes is required for drug resistance in pathogenic biofilms only [88], [89]. “Sexual” biofilms have not been described in *C. parapsilosis*, where most (and possibly all) isolates are of a single mating type [17], [90]. There are therefore aspects of the roles of Bcr1 in both species than remain to be elucidated.

Like Bcr1, Efg1 also regulates biofilm development in both *C. albicans* and *C. parapsilosis* [18], [39], [87], [91], [92]. In *C. albicans* Efg1 plays a central role in networks regulating high frequency epigenetic switching from white-to-opaque cells [93], and in filamentation of both cell types [12]. In addition, it is required for virulence [94] and for drug resistance [95]. In *C. parapsilosis*, Efg1 also regulates a high frequency switching system [18]. In both species, Efg1 directly bind to the promoters of a large number of transcription factors [18], [39]. Many of the promoters are exceptionally long, suggesting that they are regulated by several transcription factors [18], [39]. It is therefore likely that Efg1 is a member of several regulatory circuits in both species.

Although Ace2 was not identified as a component of the *C. albicans* biofilm regulatory circuit by Nobile et al [39], we and others have shown that it is required for biofilm development in this species [40], [68], which we confirmed in the current assay (Figure 2D). Ace2 regulates expression of genes during late M/early G1 phase of the cell cycle in *S. cerevisiae* and *C. albicans* [68], [96], [97]. The transcription factor is part of the RAM network (Regulation of Ace2 and Morphogenesis) that controls exit from mitosis in many fungi (reviewed in [98]). Ace2 regulates adherence of *C. albicans*, leading to sparse biofilms, and is target of Snf5, a chromatin regulator [40]. Deleting *CpACE2* results in a cell separation defect, and expression of many targets shared with *CaACE2* is reduced (e.g. *CHT1*, *CHT3*, *SCW11*) (Table S3). The function of Ace2 in regulating biofilm development in *Candida* species is therefore likely to be conserved.

Deleting the transcription factors *UME6*, *CPH2*, *CZF1* and *GZF3* reduces biofilm development in *C. parapsilosis* only (Figure 2A,D). We did not test the effect of deleting the *MKC1* kinase ortholog in *C. albicans*. It remains possible that the orthologous transcription factors may play a more minor role in biofilm growth of *C. albicans*. For example, overexpressing *UME6* enhances biofilm development in both species [48], [99]. However, we detected very little reduction in *C. albicans* biofilms formed by *ume6* deletion strains (Figure 2D, and two deletions in a different genetic background that are not shown), and it is clear that the phenotype of the *UME6* deletion in *C. parapsilosis* is considerably more severe. In *S. cerevisiae*, *UME6* is an activator of gene expression during early meiosis, whereas in *C. albicans*, the ortholog regulates filamentation. Expression of *UME6* is also increased during pseudohyphal growth in *C. parapsilosis* [99].

The *CPH2* regulon in *C. albicans* is not known, but an early microarray study suggests that *CaCPH2* regulates expression of filamentation genes, many of which are also targets of Efg1 [100]. Other regulators of filamentation, such as *NRG1*, also control biofilm formation in both *C. albicans* and *C. parapsilosis* [48]. It is likely that filamentation is more important for *C. albicans* biofilms; although both species can grow as filaments, only *C. albicans* make true hyphae, and many mutants locked in the yeast phase make poor biofilms [101], [102]. Ume6 and Cph2 may therefore have functions separate to filamentation that are important for biofilm formation in *C. parapsilosis*.

Although deleting *CZF1* does not reduce mature biofilm development in *C. albicans*, the transcription factor is required for early stage adherence [40]. The functional targets are not known [40]. In *C. parapsilosis*, *CZF1* regulates expression of transporters (Table S3); their role in biofilm development remains to be elucidated. *GZF3* encodes a GATA-type transcription factor that is induced during oxidative stress in *C. albicans* [103]. Expression is induced in biofilms, but there is no evidence that it plays a major role in *C. albicans* biofilm development [39].

### Elucidation of the biofilm transcriptome

We used RNA-seq and network analysis to characterize the transcriptome of *C. parapsilosis* biofilms. Gene sets enriched in genes downregulated in biofilms are associated with translation and with ribosome function, which probably reflects reduced growth in biofilms compared to planktonic cells (Figure 5). Genes up-regulated in *C. parapsilosis* biofilms are enriched for pathways associated with transport (including carboxylic acid transport), and are also enriched in gene sets that are upregulated in *C. albicans* biofilms [39]. A more direct comparison of the *C. parapsilosis* and *C. albicans* biofilm transcriptomes revealed that similarities between the species center on metabolic changes, and in particular monocarboxylic acid metabolism (Figure 6).

Some of the shared response may be related to the fact that biofilms are hypoxic environments [32], [63]. For example, 43 genes that are up-regulated in *C. parapsilosis* and *C. albicans* [39] biofilms and in both species in hypoxic conditions [61], [104] are enriched for processes associated with carbohydrate and lipid metabolism, which may reflect changes occurring during adaptation to hypoxic environments. Other metabolic features, such as glycerol metabolism, are also increasingly being recognized as potential virulence factors [105]. Expression of *RHR2*, glycerol-3-phosphatase is important for development of *C. albicans* biofilms, and it is suggested that glycerol levels directly regulate expression of adhesins [105]. *RHR2* expression is also increased in *C. parapsilosis* biofilms (Table S3).

Analysis of gene family enrichment was also used to compare the biofilm transcriptional response of these two species. The ATO, TES, OPT and CFEM families are enriched in both *C. parapsilosis* and *C. albicans* biofilms (Figure 6B). Although the roles of the ATO and TES families in biofilm development of *C. albicans* has not been elucidated, increased oligopeptide transfer (OPT) has been associated with early biofilm stages [106]. Some members of the CFEM family are regulated by Bcr1 in both *C. albicans* and *C. parapsilosis* [41], [44], [107]. Surprisingly, although CFEM genes are required for biofilm development in *C. albicans* [108] they do not have a similar role in *C. parapsilosis* [41]. We further confirmed this here by deleting the CFEM genes in *C. parapsilosis* in clusters (*CFEM1-4* together, *CFEM5-6* together and *CFEM7*). None of the deletion strains were defective in biofilm formation (Table S2). However, the conservation of expression of the four gene families in *C. albicans* and *C. parapsilosis* suggest that they may be suitable targets for subsequent study of core biofilm components.

Somewhat surprisingly, processes associated with adhesion and those classified as being involved in biofilm formation by the *Candida* Genome Database [109] are enriched in *C. albicans* biofilms and not in *C. parapsilosis* biofilms (Figure 6A). These may be because biofilm development in *C. albicans* is strongly correlated with the switch from yeast to hyphal growth [29]. One of the major differences between *C. parapsilosis* and *C. albicans* is that *C. parapsilosis* does not make true hyphae. Biofilms produced by *C. albicans* are composed of a mixture of yeast, pseudohyphal and hyphal cells, with the yeast cells forming the basal layer and an upper layer of hyphal cells [29]. In contrast *C. parapsilosis* biofilm contains only yeast and pseudohyphal cells in compact layers [28].

### Identification of the *C. parapsilosis* biofilm circuit

We used RNA-seq to identify the targets of the major *C. parapsilosis* transcriptional regulators during biofilm growth. The Bcr1 and Cph2 regulons are similar (Figure 7) and they have a high degree of overlap with genes enriched in the biofilm transcriptome (Figure 8). This suggests that Cph2 and Bcr1 are major biofilm regulators in *C. parapsilosis*. Both transcription factors regulate expression of genes involved in transport of carboxylic and other organic acids, and in glycolysis and monosaccharide metabolism. We observed that carboxylic acid transport or metabolism gene sets are enriched in the genes differentially regulated in many of the deletion strains (Figure 7). Even Efg1, which otherwise has little overlap with the *C. parapsilosis* biofilm network, regulates carboxylic acid transport (Figure 8C). In addition, genes that are differentially regulated in both *C. albicans* and *C. parapsilosis* biofilms are enriched in processes associated with carboxylic acid metabolism (Figure 6A). This supports our hypothesis that regulation of metabolism is important for biofilm production in both species. The shared role of Bcr1 as a biofilm regulator in the two species may therefore be related to regulation of carboxylic acid metabolism. In *C. parapsilosis* biofilms, Cph2 regulates expression of transporters and oxidoreductases, including the highly expressed *ATO* (Ammonia Transporter Outward) family. The *cph2* GSEA network is enriched for gene sets associated with *C. albicans* biofilms (Figure 8B), suggesting that *CpCPH2* may perform some of the role of the *C. albicans* biofilm regulators.

We exploited the RNA-seq data to look for evidence that the transcriptional regulators we identified work together in a network (Figure 9). We also included ChIP-seq data for Efg1 from planktonic cells [18]. We placed Efg1 at the center of the network to simplify the model, because it has many connections with the other regulators. Efg1 is also known to have many roles in both species [18], [95], [110], [111], [112], [113]. Several of the transcription factors regulate expression of other members of the putative network; some are activators (for example deleting *EFG1* reduces expression of *CZF1*) and some as repressors (deleting *CZF1* increases expression of *EFG1*). Some are linked to the putative network by only one connection (*ACE2* and *GZF3*). The role of *ACE2* in biofilm development is likely to be associated with its function in regulating cell separation [68], [97], and it may be more important for adherence rather than for mature biofilm growth [40].

It is unlikely that we have identified all the biofilm regulators in *C. parapsilosis*. For example, our transcription factor deletion collection is not complete. In addition, we have not obtained transcriptional profiling data for the *GZF3* deletion, and we have not identified the direct targets of the transcription factors (for example using ChIP-seq). We were also unable to determine the role of *NDT80*, which our GSEA analysis suggests may be involved (Figure 5). Overall, our analysis suggests that there is some degree of conservation of biofilm networks in *C. albicans* and *C. parapsilosis*. This is not surprising, considering the two species are closely related [2]. However, there are also significant differences, with four transcription factors specific to *C. parapsilosis*, and three unique to *C. albicans*. In future work, careful characterization the *C. parapsilosis* biofilm regulon will help us to elucidate the evolution of the networks in these species.

## Materials and Methods

### Strains and growth conditions

*Candida parapsilosis* strains (Table S1) were grown in YPD medium (1% yeast extract, 2% peptone, 2% glucose) at 30°C. For colony selection 2% agar was added. To select for transformants, nourseothricin (Werner Bioagents Jena, Germany) was added to YPD agar at a final concentration of 200 µg ml^−1^. Transformants containing the *LEU2* and *HIS1* markers were selected on synthetic complete (SC; 0.19% yeast nitrogen base without amino acids and ammonium sulphate, 0.5% ammonium sulphate, 2% dextrose, 0.075% mixture of amino acids, 2% agar) media without leucine or histidine. For biofilm formation, *C. parapsilosis* was grown in synthetic defined (SD) medium (0.67% yeast nitrogen base) containing 50 mM glucose. *C. albicans* was grown in Spider media (1% nutrient broth, 1% mannitol, 0.2% potassium phosphate). The media used for phenotype screening is shown in Table S2. All deletion strains were grown in 96 well plates in YPD media at 30°C overnight. The cultures were then diluted 1∶100 into a new 96 well plate containing fresh YPD media. The strains were then pinned onto agar plates using a 48 pin bolt replicator. Plates were incubated at 30°C and photographed after 2 and 3 days of growth. Each knockout was scored on growth in comparison to the control strains (CLIB214 and CPRI) on the same media, where −4 indicates a severe growth defect, −3, −2, and −1 indicate strong, moderate and marginal growth defects, 0 is similar to the control strains, and +1 is stronger growth than control strains. Scores were assigned only where the two independent replicates had the same behavior. Screens were repeated at least twice. Growth on different chemical concentrations (e.g. CuCl_2_, ketoconazole) were combined to give a single score (see Table S2). Deletion strains with interesting phenotypes were further validated by plating exact numbers of cells in decreasing concentration on test media. Control strains were included in each plate. Scores were converted to a Heatmap using Bioconductor [114].

### Construction of deletion strains in *C. parapsilosis*

To delete *LEU2* (CPAR2_805510), approximately 500 bp upstream and downstream of the open reading frame was amplified using the primer pairs CpLEU2KO1/CpLEU2KO2 and CpLEU2KO3/CpLEU2KO4 respectively (Table S6). Primers KO1 and KO2 contain recognition sites for *Kpn*I and *Apa*I respectively and KO3 and KO4 for *Sac*II and *Sac*I. The PCR products were purified using a Qiagen PCR purification kit and ligated at either end of a *SAT1* flipper cassette in pCD8 [115], generating plasmid pCpLEU2. The entire cassette plus flanking regions were excised by digestion with *Kpn*I and *Sac*I, gel purified and transformed into *C. parapsilosis* CLIB214. Integration of the cassette at *LEU2* was confirmed by PCR using a primer 5′ to *LEU2* (CpLEU2KO5) and a primer from inside the cassette (BUT237). Intact *LEU2* alleles were identified by PCR with CpLEU2KO5 and CpLEU2KO6. Primers CpLEU2KO1 and CpLEU2KO4 were used to verify recycling of the cassette and deletion of the *LEU2* gene. The same cassette was used to delete both *LEU2* alleles (Figure S1). A similar method was used to delete the *HIS1* gene (CPAR2_100200) using primers CpHIS1KO1 and CpHIS1KO2 to amplify an upstream region and primers CpHIS1KO3 and CpHIS1KO4 to amplify a downstream region. Integration of the cassette was confirmed by PCR using a primer 5′ to the gene CpHIS1KO5 and BUT237. To confirm the presence of an intact allele the primer pair CpHIS1KO5 and CpHIS1KO6 were used and primer pair CpHIS1KO1 and CpHIS1KO4 were used to verify recycling of the cassette and deletion of the *HIS1* (Figure S1). The *leu2^−^/his1^−^* strain (CPL2H1) was used as a background for all other deletion constructs.

Target genes were deleted in strain CPL2H1 using a fusion PCR method described in Noble et al [45]. Target genes are listed in Tables S1 and S2, and the primers used are listed in Table S6. Approximately 500 bp upstream and downstream of the target gene was amplified using Phusion Taq (New England BioLabs) with primer pairs 1/3 and primers 4/6. The annotated *CZF1* open reading frame was corrected (and elongated) by re-sequencing. The selectable markers, *C. dubliniensis HIS1* and *C. maltosa LEU2* genes were amplified using primers 2 (universal primer) and primer 5 from the plasmids pSN52 and pSN40 respectively. All PCR products were purified using a Qiagen PCR purification kit. The 5′ tails of primers 2 and 3 have complementary regions, as do primers 4 and 5. The 5′ PCR product, the 3′ PCR product and one of the selectable marker products were fused by PCR using primers 1 and 6. The resulting disruption cassette was transformed into the background strain (Figure 1, Figure S1). The first allele was always deleted using the *CmLEU2* selectable marker and the second allele using the *CdHIS1* gene.

Correct integration of the marker gene at the target locus was confirmed by PCR of both ends of the deletion construct; the 5′ region was confirmed using primers 5′check and either LEUcheck1/HIScheck1, and the 3′ region using primers 3′ check and either LEUcheck2/HIScheck2. Loss of the open reading frames was also confirmed using the appropriate ORF primers (Table S6).

To create the control reintegration strain, CPRI, upstream and downstream regions of the deleted *HIS1* gene were amplified using primers CpHIS1KO1/CpHIS-3 and CpHIS-4/CpHISKO7. The *Candida dulbiniensis HIS1* and *Candida maltosa LEU2* genes were amplified from the appropriate plasmids using primers 2 (universal primer) and CpHIS-5. The resulting PCR product were fused using primers CpHIS1KO1 and CpHIS1KO7 and transformed into the background strain and in turn re-introducing the *C. dulbiniensis HIS1* and *C. maltosa LEU2* genes at the original site of the *C. parapsilosis HIS1* alleles. Correct integration of the marker genes were confirmed by PCR using the primer CpHIS1KO5 and either LEUcheck1 or HIScheck1.

The *cph2*, *czf1*, *gzf3*, *ace2*, *mkc1* and *ume6* mutant strains were complemented by introducing one copy of the gene at its original site on the genome [87]. Briefly the entire ORF and promoter were amplified using the primers listed in Table S6. The resulting PCR products were digested using the enzyme sites incorporated into each primer and cloned into pSFS2a. The plasmid was then linearized within the promoter region using the relevant enzyme (*MKC1* and *UME6* (*Hpa*I), *CPH2* and *CZF1* (*Mlu*I), *GZF3* (*Bsm*BI), *ACE2* (*Bsi*WI)) and transformed into the appropriate background mutant strain. Transformants were selected on nourseothricin plates and correct integration of the plasmid was determined by PCR and using one primer upstream of the promoter and one inside the ORF (Table S6).

### Transformation of *C. parapsilosis*

Strains were transformed by electroporation as described previously with some modifications [115]. After electroporation, 950 µl of fresh YPD was added immediately and the mixture incubated at 30°C for 3–4 h. Following incubation, cells were pelleted, washed once in 1 ml of water and resuspended in 300 µl of water. 100 µl was plated onto YPD plates supplemented with nourseothricin at a concentration of 200 µg ml^−1^. Transformants were obtained following 48 h of incubation at 30°C.

The *SAT1* cassette was recycled by growing overnight in YPM (1% yeast extract, 2% peptone and 2% maltose). 100 cells were plated onto YPD plates containing 10 µg ml^−1^ of nourseothricin and incubated overnight at 30°C. Following incubation a mixture of large and small colonies were visible on the plate. Small colonies were restreaked onto fresh YPD agar plates and checked for nourseothricin sensitivity. The second allele was deleted using the same protocol.

Most deletion strains were constructed using chemical transformation. An overnight culture was diluted to an A_600_ of 0.2 in 30 ml of YPD broth. This was grown at 30°C to an A_600_ of 1. The culture was centrifuged at 4000 g for 5 min and the pellet resuspended in 3 ml of ice-cold water. The re-suspended pellet was centrifuged again as above and the pellet re-suspended in 200 µl of ice-cold TE-LiOAC (0.1M lithium acetate, 10 mM Tris and 1 mM EDTA). A transformation mix was set up that contained 10 µl of boiled and cooled salmon sperm DNA (10 mg ml^−1^), 20–30 µl of fusion PCR product and 100 µl of competent cells. This was incubated at 30°C for 30 min followed by addition of 700 µl of PLATE (0.1M lithium acetate, 10 mM Tris, 1 mM EDTA and 40% PEG 3350). Samples were incubated overnight at 30°C. Cells were heat shocked at 44°C for 15 min, centrifuged, and washed with 1 ml of YPD. The cells were centrifuged again and finally re-suspended in 100 µl of YPD followed by incubation at 30°C for 2 h. Cultures were then spread on the appropriate drop-out agar plates and incubated for 2–3 days at 30°C.

### Biofilm assays

Strains were tested for biofilm development crystal violet staining and direct observation using Nunclon Delta 24-well polystyrene plates as follows. *C. parapsilosis* trains were grown overnight at 30°C and washed twice in phosphate buffered saline (PBS), diluted to an A_600_ of 1 in SD media with 50 mM glucose, and 1 ml was added to each well. The cultures were incubated for 2 h at 37°C at 50 rpm. Wells were then washed once with 1 ml of PBS to remove non-adherent cells. 1 ml of fresh SD 50 mM glucose media was then added to each well. Plates were then incubated for 48 h at 37°C at 50 rpm. The supernatants were removed, and each well was washed twice with 1 ml of PBS. Plates were allowed to dry overnight at room temperature. Biofilms were stained with 500 µl 0.4% crystal violet for 10 min. The dye was removed and wells were washed with PBS. Plates were allowed to dry and biofilm was photographed. For *C. albicans* biofilms, 6 well plates were pre-treated with 10% fetal bovine serum overnight which was removed by washing with 1 ml of PBS. *C. albicans* strains from overnight cultures were washed twice with PBS, diluted to a starting A_600_ of 0.5 in Spider media and 5 ml was added to the pre-treated wells. The plates were incubated for 90 min at 37°C at 100 rpm. Each well was then washed once with PBS and fresh spider media was added followed by incubation for 48 h at 37°C at 100 rpm. The supernatants were removed and each well was washed twice with 1 ml of PBS.

For dry mass measurements, *C. parapsilosis* biofilms were formed in Nunclon Delta 6 well plates. Assays were set up as for the 24-well plates, except that 5 ml of media was used in each well. After the final wash, 1 ml of PBS was added to each well and adherent biofilms were scrapped from the bottom of the wells. The contents of two wells were vacuum filtered over a pre-weighed 0.8 µm nitrocellulose filter (Millipore). The filters were dried overnight in a warm room and weighed the following day. The average total biomass of each strain was calculated for 3 independent samples by subtracting the initial weight of the filter from the final weight. Statistical significance was calculated using the students two tailed paired t-test, and only those with a P-value <0.005 were retained.

### Confocal microscopy

*C. parapsilosis* biofilms were grown on Thermanox (Nunc) slides in in 6 well plates. Briefly, overnight cultures were washed twice in PBS and diluted to an A_600_ of 1 in SD media with 50 mM glucose. 5 ml was added to each well, and incubated for 2 h at 37°C at 50 rpm. The slides were gently removed and placed into a fresh well containing 5 ml of PBS, and gently placed into a well containing fresh SD/50 mM glucose media and incubated for 48 h at 37°C at 50 rpm. The slides were removed and washed as above, and then stained with 25 µg ml^−1^ concanavalin A (conA)-Alexa Fluor 594 conjugate (C-11253; Bio-science) for 45 min at 37°C. The liquid was removed from each well, and the Thermonox slides were flipped and placed on a 35-mm-diameter glass-bottomed petri dish (MatTek Corp., Ashland, MA). The biofilms were observed with a Zeiss LSM510 confocal scanning microscope with a ×40-magnification oil objective. A HeNe1 laser was used to excite at a 543-nm wavelength. All images were captured and analyzed using a Zeiss LSM Image Browser and Fiji.

### RNA-seq analysis

Biofilms for RNA-seq analysis were grown in SD media with 50 mM glucose at 37°C in Nunc 6 well plates. The biofilm was scraped from the bottom of each well and combined for RNA isolation. The number of wells combined depended on the amount of biofilm produced and the amount of biomass needed to obtain sufficient RNA for library preparation (6 wells for CLIB214 and 6–10 wells for the deletion strains). RNA was isolated using an Ambion Ribopure-Yeast RNA kit. To isolate RNA for planktonic cells, strains were grown to an A_600_ of 1 in SD media with 50 mM glucose at 37°C.

Strand specific RNA-seq library preparation and sequencing was carried out by BGI (www.genomics.cn, Hong-Kong). Paired-end reads (Illumina HiSeq 2000, 2×90 bp, 2 GB clean data) were obtained from three biological replicates from wild type (*Candida parapsilosis* CLIB214) in planktonic and biofilm conditions, and from *ace2*, *cph2*, *efg1*, *czf1*, *ume6* and *bcr1* deletion strains in biofilms. Two replicates were obtained form one deletion construct, and the third from the independent replicate strain. Samples were aligned to the genome [61] using TopHat2 [116]. HTSeq [27] was used to count mapped reads per gene. Differentially expressed genes were identified using DESeq2 [117] with an adjusted p-value threshold of 0.001 and a log_2_ fold change threshold of −1.5 and 1.5. Default parameters in DESeq2 were used, except that Cook distance filtering was turned off. Significantly differentially expressed genes were clustered using hierarchical clustering in R [114]. *C. albicans* orthologs were obtained from the Candida Gene Order Browser (CGOB) [47]. The GO term finder from Candida Genome Database [109] was used to carry out Gene Ontology analyses. All RNA-seq data is available from GEO accession number GSE57451.

### Gene family analysis

Gene families in *C. parapsilosis* and *C. albicans* were identified using Blast similarity searches [118] and the MCL algorithm [74]. In both species an inflation factor of 2.1 was used with MCL to robustly identify similar clusters. The average log_2_ fold change of significantly differentially expressed genes in each cluster was calculated, and ranked from high to low (Figure 8, Table S3). For *C. albicans* biofilms, gene expression levels were reported from a mixture of RNA-seq and microarray analysis [39]. We used significant RNA-seq values were available, and values from microarrays if the equivalent RNA-seq data was not significant. P-values for microarrays were not available. Significant genes were defined as log_2_ fold change greater than 1.5 or less than −1.5, and with an adjusted p-value less than 0.001 (for RNA-seq only). Plots were generated in R and analyzed manually.

### Gene Set Enrichment Analysis

The pre-ranked Gene Set Enrichment Analysis tool (GSEAPreRanked, see http://www.broadinstitute.org/gsea/) was used to determine gene sets that are enriched in differentially expressed genes from the RNA-seq data. Only *C. parapsilosis* genes with orthologs in *C. albicans* were included, with gene sets provided by Dr. Andre Nantel (http://www.candidagenome.org/download/community/GSEA_Nantel_2012/) [70]. Genes were ranked from highest to lowest by log2 fold change. GSEAPreRanked was used with the default options, excluding gene sets with less than 5 or more than 1000 genes, resulting in the analysis of 5249 gene sets in total. Significant results were defined as p-value lower than 0.005 and an FDR Q-value lower than 0.1. GSEAPreRanked results were further analyzed using Cytoscape (version 2.8.2) and the EnrichmentMap plugin (version 1.2, [71]) using an overlap coefficient cutoff of 0.5 and default settings. The size of the nodes correlates to the number of genes in the gene sets from Sellam et al [70], which are based on *C. albicans*. We use a background of *C. parapsilosis* orthologs for the GSEA, but the networks generated in Cytoscape reflect the original gene sets from Sellam et al [70].

## Supporting Information

Dataset S1 Cytoscape data for Gene Set Enrichment Analysis.
(ZIP)

Figure S1 Construction of gene deletions and reintroduction of wildtype genes in *C. parapsilosis*. (A) The *C. parapsilosis leu2-/his1-* strain (CPL2H1) was created in CLIB214 using the *SAT1* flipper method as described in Ding and Butler [35]. (B) A fusion PCR method was used to delete genes by replacement with either the *Candida maltosa LEU2* gene or the *Candida dublinensis HIS1* gene in *C. parapsilosis* CPL2H1. (C) Re-introduction of a wildtype allele of the deleted gene (YFG) using the plasmid pSFS2A. All constructs were confirmed by PCR using the indicated primers.
(EPS)

Figure S2 GSEA analysis of transcriptional profile of *C. parapsilosis* biofilms compared to planktonic cells (from Figure 5). Gene sets enriched (GSEAPreRanked) in genes up-regulated in biofilm conditions are shown in red, and in down-regulated genes are shown in blue. The size of nodes represents the number of genes in the gene set. Edges are drawn when genes between two gene sets overlap. The Cytoscape plugin EnrichmentMap was used to visualize the network with default parameters. Nodes with one edge or less are shown separately. Gene set names are from http://www.candidagenome.org/download/community/GSEA_Nantel_2012/. Cytoscape data is available in Dataset S1.
(EPS)

Figure S3 Comparison of gene sets enriched in the *C. parapsilosis* biofilm transcriptome and in (A) *bcr1* (B) *cph2* and (C) *efg1* deletion strains (from Figure 8).
(EPS)

Table S1 List of strains used.
(XLS)

Table S2 Phenotype scores for *C. parapsilosis* and *C. albicans* gene deletion strains.
(XLS)

Table S3 RNA-seq profiling of *C. parapsilosis* biofilms.
(XLS)

Table S4 GO-term enrichment for RNA-seq experiments.
(XLS)

Table S5 Gene family enrichment, as shown in Fig. 6B.
(XLS)

Table S6 List of primers used.
(XLS)

Text S1 Describes phenotype screen of additional *C. parapsilosis* gene deletions and comparison of copper and iron regulation in *C. parapsilosis* and *C. albicans*.
(DOCX)

## References

[1] Lachance M-A, Boekhout T, Scorzetti G, Fell JW, Kurtzmann CP (2011) *Candida* Berkhout (1923). In: Kurtzman CP, Fell JW, Boekhout T, editors. The Yeasts, a Taxonomic Study. 5th ed. Amsterdam: Elsevier. pp. 987–1278.

[2] Fitzpatrick DA, Logue ME, Stajich JE, Butler G (2006) A fungal phylogeny based on 42 complete genomes derived from supertree and combined gene analysis. BMC Evol Biol 6: 99. 17121679 10.1186/1471-2148-6-99 PMC1679813

[3] Wong S, Fares MA, Zimmermann W, Butler G, Wolfe KH (2003) Evidence from comparative genomics for a complete sexual cycle in the ‘asexual’ pathogenic yeast *Candida glabrata* . Genome Biol 4: R10. 12620120 10.1186/gb-2003-4-2-r10 PMC151300

[4] Seervai RN, Jones SK Jr, Hirakawa MP, Porman AM, Bennett RJ (2013) Parasexuality and ploidy change in *Candida tropicalis* . Eukaryot Cell 12: 1629–1640. 24123269 10.1128/EC.00128-13 PMC3889571

[5] Hickman MA, Zeng G, Forche A, Hirakawa MP, Abbey D, et al (2013) The ‘obligate diploid’ *Candida albicans* forms mating-competent haploids. Nature 494: 55–59. 23364695 10.1038/nature11865 PMC3583542

[6] Sherwood RK, Scaduto CM, Torres SE, Bennett RJ (2014) Convergent evolution of a fused sexual cycle promotes the haploid lifestyle. Nature 506: 387–390. 24390351 10.1038/nature12891 PMC4051440

[7] Santos MA, Gomes AC, Santos MC, Carreto LC, Moura GR (2011) The genetic code of the fungal CTG clade. C R Biol 334: 607–611. 21819941 10.1016/j.crvi.2011.05.008

[8] Lass-Florl C (2009) The changing face of epidemiology of invasive fungal disease in Europe. Mycoses 52: 197–205. 19391253 10.1111/j.1439-0507.2009.01691.x

[9] Pfaller MA, Diekema DJ (2007) Epidemiology of invasive candidiasis: a persistent public health problem. Clin Microbiol Rev 20: 133–163. 17223626 10.1128/CMR.00029-06 PMC1797637

[10] Pfaller MA, Castanheira M, Messer SA, Moet GJ, Jones RN (2010) Variation in *Candida* spp. distribution and antifungal resistance rates among bloodstream infection isolates by patient age: report from the SENTRY Antimicrobial Surveillance Program (2008–2009). Diagn Microbiol Infect Dis 68: 278–283. 20846808 10.1016/j.diagmicrobio.2010.06.015

[11] Mayer FL, Wilson D, Hube B (2013) *Candida albicans* pathogenicity mechanisms. Virulence 4: 119–128. 23302789 10.4161/viru.22913 PMC3654610

[12] Si H, Hernday AD, Hirakawa MP, Johnson AD, Bennett RJ (2013) *Candida albicans* white and opaque cells undergo distinct programs of filamentous growth. PLoS Pathog 9: e1003210. 23505370 10.1371/journal.ppat.1003210 PMC3591317

[13] Porman AM, Hirakawa MP, Jones SK, Wang N, Bennett RJ (2013) MTL-independent phenotypic switching in *Candida tropicalis* and a dual role for Wor1 in regulating switching and filamentation. PLoS Genet 9: e1003369. 23555286 10.1371/journal.pgen.1003369 PMC3605238

[14] Xie J, Du H, Guan G, Tong Y, Kourkoumpetis TK, et al (2012) N-Acetylglucosamine induces White-to-Opaque switching and mating in *Candida tropicalis*, providing new insights into adaptation and fungal sexual evolution. Eukaryot Cell 11: 773–782. 22544905 10.1128/EC.00047-12 PMC3370467

[15] Pammi M, Holland L, Butler G, Gacser A, Bliss JM (2013) *Candida parapsilosis* is a significant neonatal pathogen: a systematic review and meta-analysis. Pediatr Infect Dis J 32: e206–216. 23340551 10.1097/INF.0b013e3182863a1c PMC3681839

[16] Butler G (2010) Fungal sex and pathogenesis. Clin Microbiol Rev 23: 140–159. 20065328 10.1128/CMR.00053-09 PMC2806657

[17] Sai S, Holland L, McGee CF, Lynch DB, Butler G (2011) Evolution of mating within the *Candida parapsilosis* species group. Eukaryot Cell 10: 578–587. 21335529 10.1128/EC.00276-10 PMC3127640

[18] Connolly LA, Riccombeni A, Grozer Z, Holland LM, Lynch DB, et al (2013) The APSES transcription factor Efg1 is a global regulator that controls morphogenesis and biofilm formation in *Candida parapsilosis* . Mol Microbiol 90: 36–53. 23895281 10.1111/mmi.12345 PMC3912905

[19] Barchiesi F, Caggiano G, Falconi Di Francesco L, Montagna MT, Barbuti S, et al (2004) Outbreak of fungemia due to *Candida parapsilosis* in a pediatric oncology unit. Diagn Microbiol Infect Dis 49: 269–271. 15313532 10.1016/j.diagmicrobio.2004.03.011

[20] Clark TA, Slavinski SA, Morgan J, Lott T, Arthington-Skaggs BA, et al (2004) Epidemiologic and molecular characterization of an outbreak of *Candida parapsilosis* bloodstream infections in a community hospital. J Clin Microbiol 42: 4468–4472. 15472295 10.1128/JCM.42.10.4468-4472.2004 PMC522355

[21] DiazGranados CA, Martinez A, Deaza C, Valderrama S (2008) An outbreak of *Candida* spp. bloodstream infection in a tertiary care center in Bogota, Colombia. Braz J Infect Dis 12: 390–394. 19219278 10.1590/s1413-86702008000500009

[22] Dizbay M, Kalkanci A, Sezer BE, Aktas F, Aydogan S, et al (2008) Molecular investigation of a fungemia outbreak due to *Candida parapsilosis* in an intensive care unit. Braz J Infect Dis 12: 395–399. 19219279 10.1590/s1413-86702008000500010

[23] Levin AS, Costa SF, Mussi NS, Basso M, Sinto SI, et al (1998) *Candida parapsilosis* fungemia associated with implantable and semi-implantable central venous catheters and the hands of healthcare workers. Diagn Microbiol Infect Dis 30: 243–249. 9582583 10.1016/s0732-8893(98)00006-6

[24] van Asbeck EC, Huang YC, Markham AN, Clemons KV, Stevens DA (2007) *Candida parapsilosis* fungemia in neonates: genotyping results suggest healthcare workers hands as source, and review of published studies. Mycopathologia 164: 287–293. 17874281 10.1007/s11046-007-9054-3

[25] Almirante B, Rodriguez D, Cuenca-Estrella M, Almela M, Sanchez F, et al (2006) Epidemiology, risk factors, and prognosis of *Candida parapsilosis* bloodstream infections: case-control population-based surveillance study of patients in Barcelona, Spain, from 2002 to 2003. J Clin Microbiol 44: 1681–1685. 16672393 10.1128/JCM.44.5.1681-1685.2006 PMC1479182

[26] Clerihew L, Lamagni TL, Brocklehurst P, McGuire W (2007) *Candida parapsilosis* infection in very low birthweight infants. Arch Dis Child Fetal Neonatal Ed 92: F127–129. 17337658 10.1136/fnn.2006.097758 PMC2675456

[27] Kuhn DM, Chandra J, Mukherjee PK, Ghannoum MA (2002) Comparison of biofilms formed by *Candida albicans* and *Candida parapsilosis* on bioprosthetic surfaces. Infect Immun 70: 878–888. 11796623 10.1128/iai.70.2.878-888.2002 PMC127692

[28] Silva S, Henriques M, Martins A, Oliveira R, Williams D, et al (2009) Biofilms of non-*Candida albicans Candida* species: quantification, structure and matrix composition. Med Mycol 47: 681–689. 19888800 10.3109/13693780802549594

[29] Blankenship JR, Mitchell AP (2006) How to build a biofilm: a fungal perspective. Curr Opin Microbiol 9: 588–594. 17055772 10.1016/j.mib.2006.10.003

[30] Seneviratne CJ, Jin L, Samaranayake LP (2008) Biofilm lifestyle of *Candida*: a mini review. Oral Dis 14: 582–590. 19076549 10.1111/j.1601-0825.2007.01424.x

[31] Ramage G, Mowat E, Jones B, Williams C, Lopez-Ribot J (2009) Our current understanding of fungal biofilms. Crit Rev Microbiol 35: 340–355. 19863383 10.3109/10408410903241436

[32] Sellam A, Al-Niemi T, McInnerney K, Brumfield S, Nantel A, et al (2009) A *Candida albicans* early stage biofilm detachment event in rich medium. BMC Microbiol 9: 25. 19187560 10.1186/1471-2180-9-25 PMC2647545

[33] Pannanusorn S, Fernandez V, Romling U (2013) Prevalence of biofilm formation in clinical isolates of *Candida* species causing bloodstream infection. Mycoses 56: 264–272. 23113805 10.1111/myc.12014

[34] Kuhn DM, Chandra J, Mukherjee PK, Ghannoum MA (2002) Comparison of biofilms formed by *Candida albicans* and *Candida parapsilosis* on bioprosthetic surfaces. Infect Immun 70: 878–888. 11796623 10.1128/iai.70.2.878-888.2002 PMC127692

[35] Ding C, Butler G (2007) Development of a gene knockout system in *Candida parapsilosis* reveals a conserved role for *BCR1* in biofilm formation. Eukaryot Cell 6: 1310–1319. 17586721 10.1128/EC.00136-07 PMC1951126

[36] Gacser A, Trofa D, Schafer W, Nosanchuk JD (2007) Targeted gene deletion in *Candida parapsilosis* demonstrates the role of secreted lipase in virulence. J Clin Invest 117: 3049–3058. 17853941 10.1172/JCI32294 PMC1974868

[37] Finkel JS, Mitchell AP (2011) Genetic control of *Candida albicans* biofilm development. Nature Rev Microbiol 9: 109–118. 21189476 10.1038/nrmicro2475 PMC3891587

[38] Nobile CJ, Mitchell AP (2006) Genetics and genomics of *Candida albicans* biofilm formation. Cell Microbiol 8: 1382–1391. 16848788 10.1111/j.1462-5822.2006.00761.x

[39] Nobile CJ, Fox EP, Nett JE, Sorrells TR, Mitrovich QM, et al (2012) A recently evolved transcriptional network controls biofilm development in *Candida albicans* . Cell 148: 126–138. 22265407 10.1016/j.cell.2011.10.048 PMC3266547

[40] Finkel JS, Xu W, Huang D, Hill EM, Desai JV, et al (2012) Portrait of *Candida albicans* adherence regulators. PLoS Pathog 8: e1002525. 22359502 10.1371/journal.ppat.1002525 PMC3280983

[41] Ding C, Vidanes GM, Maguire SL, Guida A, Synnott JM, et al (2011) Conserved and divergent roles of Bcr1 and CFEM proteins in *Candida parapsilosis* and *Candida albicans* . PLoS ONE 6: e28151. 22145027 10.1371/journal.pone.0028151 PMC3228736

[42] Davis DA, Bruno VM, Loza L, Filler SG, Mitchell AP (2002) *Candida albicans* Mds3p, a conserved regulator of pH responses and virulence identified through insertional mutagenesis. Genetics 162: 1573–1581. 12524333 10.1093/genetics/162.4.1573 PMC1462392

[43] Homann OR, Dea J, Noble SM, Johnson AD (2009) A phenotypic profile of the *Candida albicans* regulatory network. PLoS Genet 5: e1000783. 20041210 10.1371/journal.pgen.1000783 PMC2790342

[44] Nobile CJ, Mitchell AP (2005) Regulation of cell-surface genes and biofilm formation by the *C. albicans* transcription factor Bcr1p. Curr Biol 15: 1150–1155. 15964282 10.1016/j.cub.2005.05.047

[45] Noble SM, Johnson AD (2005) Strains and strategies for large-scale gene deletion studies of the diploid human fungal pathogen *Candida albicans* . Eukaryot Cell 4: 298–309. 15701792 10.1128/EC.4.2.298-309.2005 PMC549318

[46] Fitzpatrick DA, O'Gaora P, Byrne KP, Butler G (2010) Analysis of gene evolution and metabolic pathways using the Candida Gene Order Browser. BMC Genomics 11: 290. 20459735 10.1186/1471-2164-11-290 PMC2880306

[47] Maguire SL, Oheigeartaigh SS, Byrne KP, Schroder MS, O'Gaora P, et al (2013) Comparative genome analysis and gene finding in *Candida* species using CGOB. Mol Biol Evol 30: 1281–1291. 23486613 10.1093/molbev/mst042 PMC3649674

[48] Banerjee M, Thompson DS, Lazzell A, Carlisle PL, Pierce C, et al (2008) *UME6*, a novel filament-specific regulator of *Candida albicans* hyphal extension and virulence. Mol Biol Cell 19: 1354–1365. 18216277 10.1091/mbc.E07-11-1110 PMC2291399

[49] Alonso-Monge R, Navarro-Garcia F, Roman E, Negredo AI, Eisman B, et al (2003) The Hog1 mitogen-activated protein kinase is essential in the oxidative stress response and chlamydospore formation in *Candida albicans* . Eukaryot Cell 2: 351–361. 12684384 10.1128/EC.2.2.351-361.2003 PMC154845

[50] Baek YU, Li M, Davis DA (2008) *Candida albicans* ferric reductases are differentially regulated in response to distinct forms of iron limitation by the Rim101 and CBF transcription factors. Eukaryot Cell 7: 1168–1179. 18503007 10.1128/EC.00108-08 PMC2446673

[51] Chen C, Pande K, French SD, Tuch BB, Noble SM (2011) An iron homeostasis regulatory circuit with reciprocal roles in *Candida albicans* commensalism and pathogenesis. Cell Host Microbe 10: 118–135. 21843869 10.1016/j.chom.2011.07.005 PMC3165008

[52] Hoot SJ, Oliver BG, White TC (2008) *Candida albicans UPC2* is transcriptionally induced in response to antifungal drugs and anaerobicity through Upc2p-dependent and -independent mechanisms. Microbiology 154: 2748–2756. 18757808 10.1099/mic.0.2008/017475-0 PMC2577385

[53] Synnott JM, Guida A, Mulhern-Haughey S, Higgins DG, Butler G (2010) Regulation of the hypoxic response in *Candida albicans* . Eukaryot Cell 9: 1734–1746. 20870877 10.1128/EC.00159-10 PMC2976306

[54] Drakulic T, Temple MD, Guido R, Jarolim S, Breitenbach M, et al (2005) Involvement of oxidative stress response genes in redox homeostasis, the level of reactive oxygen species, and ageing in *Saccharomyces cerevisiae* . FEMS Yeast Res 5: 1215–1228. 16087409 10.1016/j.femsyr.2005.06.001

[55] Zhao H, Eide DJ (1997) Zap1p, a metalloregulatory protein involved in zinc-responsive transcriptional regulation in *Saccharomyces cerevisiae* . Mol Cell Biol 17: 5044–5052. 9271382 10.1128/mcb.17.9.5044 PMC232355

[56] Thiele DJ (1988) *ACE1* regulates expression of the *Saccharomyces cerevisiae* metallothionein gene. Mol Cell Biol 8: 2745–2752. 3043194 10.1128/mcb.8.7.2745-2752.1988 PMC363491

[57] Olesen JT, Guarente L (1990) The *HAP2* subunit of yeast CCAAT transcriptional activator contains adjacent domains for subunit association and DNA recognition: model for the HAP2/3/4 complex. Genes Dev 4: 1714–1729. 2123465 10.1101/gad.4.10.1714

[58] Lamb TM, Mitchell AP (2003) The transcription factor Rim101p governs ion tolerance and cell differentiation by direct repression of the regulatory genes NRG1 and SMP1 in *Saccharomyces cerevisiae* . Mol Cell Biol 23: 677–686. 12509465 10.1128/MCB.23.2.677-686.2003 PMC151549

[59] Vik A, Rine J (2001) Upc2p and Ecm22p, dual regulators of sterol biosynthesis in *Saccharomyces cerevisiae* . Mol Cell Biol 21: 6395–6405. 11533229 10.1128/MCB.21.19.6395-6405.2001 PMC99787

[60] Chen C, Pande K, French SD, Tuch BB, Noble SM (2011) An iron homeostasis regulatory circuit with reciprocal roles in *Candida albicans* commensalism and pathogenesis. Cell Host Microbe 10: 118–135. 21843869 10.1016/j.chom.2011.07.005 PMC3165008

[61] Guida A, Lindstadt C, Maguire SL, Ding C, Higgins DG, et al (2011) Using RNA-seq to determine the transcriptional landscape and the hypoxic response of the pathogenic yeast *Candida parapsilosis* . BMC Genomics 12: 628. 22192698 10.1186/1471-2164-12-628 PMC3287387

[62] MacPherson S, Akache B, Weber S, De Deken X, Raymond M, et al (2005) *Candida albicans* zinc cluster protein Upc2p confers resistance to antifungal drugs and is an activator of ergosterol biosynthetic genes. Antimicrob Agents Chemother 49: 1745–1752. 15855491 10.1128/AAC.49.5.1745-1752.2005 PMC1087678

[63] Rossignol T, Ding C, Guida A, d'Enfert C, Higgins DG, et al (2009) Correlation between biofilm formation and the hypoxic response in *Candida parapsilosis* . Eukaryot Cell 8: 550–559. 19151323 10.1128/EC.00350-08 PMC2669199

[64] Weissman Z, Kornitzer D (2004) A family of *Candida* cell surface haem-binding proteins involved in haemin and haemoglobin-iron utilization. Mol Microbiol 53: 1209–1220. 15306022 10.1111/j.1365-2958.2004.04199.x

[65] Weissman Z, Shemer R, Conibear E, Kornitzer D (2008) An endocytic mechanism for haemoglobin-iron acquisition in *Candida albicans* . Mol Microbiol 69: 201–217. 18466294 10.1111/j.1365-2958.2008.06277.x

[66] Puig S, Askeland E, Thiele DJ (2005) Coordinated remodeling of cellular metabolism during iron deficiency through targeted mRNA degradation. Cell 120: 99–110. 15652485 10.1016/j.cell.2004.11.032

[67] Puig S, Vergara SV, Thiele DJ (2008) Cooperation of two mRNA-binding proteins drives metabolic adaptation to iron deficiency. Cell Metab 7: 555–564. 18522836 10.1016/j.cmet.2008.04.010 PMC2459314

[68] Kelly MT, MacCallum DM, Clancy SD, Odds FC, Brown AJ, et al (2004) The *Candida albicans CaACE2* gene affects morphogenesis, adherence and virulence. Mol Microbiol 53: 969–983. 15255906 10.1111/j.1365-2958.2004.04185.x

[69] Andes D, Nett J, Oschel P, Albrecht R, Marchillo K, et al (2004) Development and characterization of an in vivo central venous catheter *Candida albicans* biofilm model. Infect Immun 72: 6023–6031. 15385506 10.1128/IAI.72.10.6023-6031.2004 PMC517581

[70] Sellam A, van Het Hoog M, Tebbji F, Beaurepaire C, Whiteway M, et al (2014) Modeling the transcriptional regulatory network that controls the early hypoxic response in *Candida albicans* . Eukaryot Cell 13: 675–90 doi: 10.1128/EC.00292-13 24681685 10.1128/EC.00292-13 PMC4060469

[71] Merico D, Isserlin R, Stueker O, Emili A, Bader GD (2010) Enrichment map: a network-based method for gene-set enrichment visualization and interpretation. PLoS ONE 5: e13984. 21085593 10.1371/journal.pone.0013984 PMC2981572

[72] Garcia-Sanchez S, Aubert S, Iraqui I, Janbon G, Ghigo JM, et al (2004) *Candidaalbicans* biofilms: a developmental state associated with specific and stable gene expression patterns. Eukaryot Cell 3: 536–545. 15075282 10.1128/EC.3.2.536-545.2004 PMC387656

[73] Butler G, Lorenz M, Gow NAR (2012) Evolution and genomics of the pathogenic *Candida* species complex. In: Sibley DL, Howlett BK, Heitman J, editors. Evolution of virulence in eukaryotic microbes: John Wiley & Sons, Inc. pp. 404–421.

[74] Enright AJ, Van Dongen S, Ouzounis CA (2002) An efficient algorithm for large-scale detection of protein families. Nucleic Acids Res 30: 1575–1584. 11917018 10.1093/nar/30.7.1575 PMC101833

[75] Palkova Z, Devaux F, Icicova M, Minarikova L, Le Crom S, et al (2002) Ammonia pulses and metabolic oscillations guide yeast colony development. Mol Biol Cell 13: 3901–3914. 12429834 10.1091/mbc.E01-12-0149 PMC133602

[76] Lubkowitz MA, Hauser L, Breslav M, Naider F, Becker JM (1997) An oligopeptide transport gene from *Candida albicans* . Microbiology 143: 387–396. 9043116 10.1099/00221287-143-2-387

[77] Cao YY, Cao YB, Xu Z, Ying K, Li Y, et al (2005) cDNA microarray analysis of differential gene expression in Candida albicans biofilm exposed to farnesol. Antimicrob Agents Chemother 49: 584–589. 15673737 10.1128/AAC.49.2.584-589.2005 PMC547270

[78] De Groot PW, Hellingwerf KJ, Klis FM (2003) Genome-wide identification of fungal GPI proteins. Yeast 20: 781–796. 12845604 10.1002/yea.1007

[79] Maguire SL, Wang C, Holland LM, Brunel F, Neuveglise C, et al (2014) Zinc finger transcription factors displaced SREBP proteins as the major sterol regulators during Saccharomycotina evolution. PLoS Genet 10: e1004076. 24453983 10.1371/journal.pgen.1004076 PMC3894159

[80] Lane S, Birse C, Zhou S, Matson R, Liu H (2001) DNA array studies demonstrate convergent regulation of virulence factors by Cph1, Cph2, and Efg1 in *Candida albicans* . J Biol Chem 276: 48988–48996. 11595734 10.1074/jbc.M104484200

[81] Lane S, Zhou S, Pan T, Dai Q, Liu H (2001) The basic helix-loop-helix transcription factor Cph2 regulates hyphal development in *Candida albicans* partly via *TEC1* . Mol Cell Biol 21: 6418–6428. 11533231 10.1128/MCB.21.19.6418-6428.2001 PMC99789

[82] Bien CM, Espenshade PJ (2010) Sterol regulatory element binding proteins in fungi: hypoxic transcription factors linked to pathogenesis. Eukaryot Cell 9: 352–359. 20118213 10.1128/EC.00358-09 PMC2837984

[83] Butler G (2013) Hypoxia and gene expression in eukaryotic microbes. Ann Rev Microbiol 67: 291–312. 23808338 10.1146/annurev-micro-092412-155658

[84] Di Rienzi SC, Collingwood D, Raghurama MK, Brewer BJ (2009) Fragile genomic sites are associated with origins of replication. Gen Biol Evol 2009: 350–363. 10.1093/gbe/evp034 PMC2817429 20333204

[85] Nelissen B, De Wachter R, Goffeau A (1997) Classification of all putative permeases and other membrane plurispanners of the major facilitator superfamily encoded by the complete genome of *Saccharomyces cerevisiae* . FEMS Microbiol Rev 21: 113–134. 9348664 10.1111/j.1574-6976.1997.tb00347.x

[86] Pannanusorn S, Ramirez-Zavala B, Lunsdorf H, Agerberth B, Morschhauser J, et al (2014) Characterization of biofilm formation and the role of *BCR1* in clinical isolates of *Candida parapsilosis* . Eukaryot Cell 13: 438–451. 24297446 10.1128/EC.00181-13 PMC4000102

[87] Lin CH, Kabrawala S, Fox EP, Nobile CJ, Johnson AD, et al (2013) Genetic control of conventional and pheromone-stimulated biofilm formation in *Candida albicans* . PLoS Pathog 9: e1003305. 23637598 10.1371/journal.ppat.1003305 PMC3630098

[88] Srikantha T, Daniels KJ, Pujol C, Kim E, Soll DR (2013) Identification of genes upregulated by the transcription factor Bcr1 that are involved in impermeability, impenetrability, and drug resistance of *Candida albicans* a/alpha biofilms. Eukaryot Cell 12: 875–888. 23563485 10.1128/EC.00071-13 PMC3675989

[89] Yi S, Sahni N, Daniels KJ, Lu KL, Srikantha T, et al (2011) Alternative Mating Type Configurations (a/alpha versus a/a or alpha/alpha) of Candida albicans Result in Alternative Biofilms Regulated by Different Pathways. PLoS Biol 9: e1001117. 21829325 10.1371/journal.pbio.1001117 PMC3149048

[90] Logue ME, Wong S, Wolfe KH, Butler G (2005) A genome sequence survey shows that the pathogenic yeast *Candida parapsilosis* has a defective *MTLa1* allele at its mating type locus. Eukaryot Cell 4: 1009–1017. 15947193 10.1128/EC.4.6.1009-1017.2005 PMC1151992

[91] Ramage G, Saville SP, Thomas DP, Lopez-Ribot JL (2005) *Candida* biofilms: an update. Eukaryot Cell 4: 633–638. 15821123 10.1128/EC.4.4.633-638.2005 PMC1087806

[92] Stichternoth C, Ernst JF (2009) Hypoxic adaptation by Efg1 regulates biofilm formation of *Candida albicans* . Appl Environ Microbiol 3663–3672. 19346360 10.1128/AEM.00098-09 PMC2687269

[93] Zordan RE, Miller MG, Galgoczy DJ, Tuch BB, Johnson AD (2007) Interlocking transcriptional feedback loops control White-Opaque switching in *Candida albicans* . PLoS Biol 5: e256. 17880264 10.1371/journal.pbio.0050256 PMC1976629

[94] Lo HJ, Kohler JR, DiDomenico B, Loebenberg D, Cacciapuoti A, et al (1997) Nonfilamentous *C. albicans* mutants are avirulent. Cell 90: 939–949. 9298905 10.1016/s0092-8674(00)80358-x

[95] Bink A, Govaert G, Vandenbosch D, Kucharikova S, Coenye T, et al (2012) Transcription factor Efg1 contributes to the tolerance of *Candida albicans* biofilms against antifungal agents *in vitro* and *in vivo* . J Med Microbiol 61: 813–819. 22422573 10.1099/jmm.0.041020-0

[96] Dohrmann PR, Butler G, Tamai K, Dorland S, Greene JR, et al (1992) Parallel pathways of gene regulation: homologous regulators *SWI5* and *ACE2* differentially control transcription of *HO* and chitinase. Genes Dev 6: 93–104. 1730413 10.1101/gad.6.1.93

[97] Doolin MT, Johnson AL, Johnston LH, Butler G (2001) Overlapping and distinct roles of the duplicated yeast transcription factors Ace2p and Swi5p. Mol Microbiol 40: 422–432. 11309124 10.1046/j.1365-2958.2001.02388.x

[98] Saputo S, Chabrier-Rosello Y, Luca FC, Kumar A, Krysan DJ (2012) The RAM network in pathogenic fungi. Eukaryot Cell 11: 708–717. 22544903 10.1128/EC.00044-12 PMC3370468

[99] Lackey E, Vipulanandan G, Childers DS, Kadosh D (2013) Comparative evolution of morphological regulatory functions in *Candida* species. Eukaryot Cell 12: 1356–1368. 23913541 10.1128/EC.00164-13 PMC3811340

[100] Lane S, Birse C, Zhou S, Matson R, Liu H (2001) DNA array studies demonstrate convergent regulation of virulence factors by Cph1, Cph2, and Efg1 in *Candida albicans* . J Biol Chem 276: 48988–48996. 11595734 10.1074/jbc.M104484200

[101] Thompson DS, Carlisle PL, Kadosh D (2011) Coevolution of morphology and virulence in *Candida* species. Eukaryot Cell 10: 1173–1182. 21764907 10.1128/EC.05085-11 PMC3187052

[102] Ren D, Bedzyk LA, Thomas SM, Ye RW, Wood TK (2004) Gene expression in *Escherichia coli* biofilms. Appl Microbiol Biotechnol 64: 515–524. 14727089 10.1007/s00253-003-1517-y

[103] Wang Y, Cao YY, Jia XM, Cao YB, Gao PH, et al (2006) Cap1p is involved in multiple pathways of oxidative stress response in *Candida albicans* . Free Radic Biol Med 40: 1201–1209. 16545688 10.1016/j.freeradbiomed.2005.11.019

[104] Setiadi ER, Doedt T, Cottier F, Noffz C, Ernst JF (2006) Transcriptional response of *Candida albicans* to hypoxia: linkage of oxygen sensing and Efg1p-regulatory networks. J Mol Biol 361: 399–411. 16854431 10.1016/j.jmb.2006.06.040

[105] Desai JV, Bruno VM, Ganguly S, Stamper RJ, Mitchell KF, et al (2013) Regulatory role of glycerol in *Candida albicans* biofilm formation. MBio 4: e00637–00612. 23572557 10.1128/mBio.00637-12 PMC3622937

[106] Yeater KM, Chandra J, Cheng G, Mukherjee PK, Zhao X, et al (2007) Temporal analysis of *Candida albicans* gene expression during biofilm development. Microbiology 153: 2373–2385. 17660402 10.1099/mic.0.2007/006163-0

[107] Nobile CJ, Andes DR, Nett JE, Smith FJ, Yue F, et al (2006) Critical role of Bcr1-dependent adhesins in *C. albicans* biofilm formation *in vitro* and *in vivo* . PLoS Pathog 2: e63. 16839200 10.1371/journal.ppat.0020063 PMC1487173

[108] Perez A, Pedros B, Murgui A, Casanova M, Lopez-Ribot JL, et al (2006) Biofilm formation by *Candida albicans* mutants for genes coding fungal proteins exhibiting the eight-cysteine-containing CFEM domain. FEMS Yeast Res 6: 1074–1084. 17042757 10.1111/j.1567-1364.2006.00131.x

[109] Inglis DO, Arnaud MB, Binkley J, Shah P, Skrzypek MS, et al (2012) The Candida genome database incorporates multiple *Candida* species: multispecies search and analysis tools with curated gene and protein information for *Candida albicans* and *Candida glabrata* . Nucleic Acids Res 40: D667–674. 22064862 10.1093/nar/gkr945 PMC3245171

[110] Braun BR, Johnson AD (2000) *TUP1*, *CPH1* and *EFG1* make independent contributions to filamentation in *Candida albicans* . Genetics 155: 57–67. 10790384 10.1093/genetics/155.1.57 PMC1461068

[111] Doedt T, Krishnamurthy S, Bockmuhl DP, Tebarth B, Stempel C, et al (2004) APSES proteins regulate morphogenesis and metabolism in *Candida albicans* . Mol Biol Cell 15: 3167–3180. 15218092 10.1091/10.1091/mbc.E03-11-0782 PMC452574

[112] Liu H (2001) Transcriptional control of dimorphism in *Candida albicans* . Curr Opin Microbiol 4: 728–735. 11731326 10.1016/s1369-5274(01)00275-2

[113] Sohn K, Urban C, Brunner H, Rupp S (2003) EFG1 is a major regulator of cell wall dynamics in *Candida albicans* as revealed by DNA microarrays. Mol Microbiol 47: 89–102. 12492856 10.1046/j.1365-2958.2003.03300.x

[114] Gentleman RC, Carey VJ, Bates DM, Bolstad B, Dettling M, et al (2004) Bioconductor: open software development for computational biology and bioinformatics. Genome Biol 5: R80. 15461798 10.1186/gb-2004-5-10-r80 PMC545600

[115] Ding C, Butler G (2007) Development of a gene knockout system in *Candida parapsilosis* reveals a conserved role for *BCR1* in biofilm formation. Eukaryot Cell 6: 1310–1319. 17586721 10.1128/EC.00136-07 PMC1951126

[116] Kim D, Pertea G, Trapnell C, Pimentel H, Kelley R, et al (2013) TopHat2: accurate alignment of transcriptomes in the presence of insertions, deletions and gene fusions. Genome Biol 14: R36. 23618408 10.1186/gb-2013-14-4-r36 PMC4053844

[117] Anders S, Huber W (2010) Differential expression analysis for sequence count data. Genome Biol 11: R106. 20979621 10.1186/gb-2010-11-10-r106 PMC3218662

[118] Altschul SF, Gish W, Miller W, Myers EW, Lipman DJ (1990) Basic local alignment search tool. J Mol Biol 215: 403–410. 2231712 10.1016/S0022-2836(05)80360-2

