# Supplementary material for: Comparative Phenotypic Analysis of the Major Fungal Pathogens Candida parapsilosis and Candida albicans
Source: PLoS Pathog. 2014 Sep 18;10(9):e1004365. doi: 10.1371/journal.ppat.1004365 (PMC4169492; doi:10.1371/journal.ppat.1004365)
Supplement: Text S1 — Describes phenotype screen of additional C. parapsilosis gene deletions and comparison of copper and iron regulation in C. parapsilosis and C. albicans. (DOCX) [file ppat.1004365.s011.docx]

**Text S1**

*Phenotype screen of additional C. parapsilosis gene deletions***.**

We deleted nine transcription factors in *C. parapsilosis* that are not represented in the *C. albicans* collection from Homann et al [1] (*ADA2, BRE1, CAS1, CPH1, FLO8, RFG1, SIZ1, UME6* and *WOR1*). The ADA2 deletion has a growth defect and was therefore not included in the phenotype screen. Deleting *CAS1*, *CPH1, FLO8, SIZ1, UME6* or *WOR1* has no effects in the conditions tested (Table S2). Deleting *BRE1* has pleiotropic effects, with reduced growth on acid, alkali, BPS, copper, Congo red, SDS and caspofungin (Figure 1B). Very little is known about the function of *BRE1* in *C. albicans*, except that it regulates filamentation [2]. Deleting *RFG1* in *C. parapsilosis* confers sensitivity to caspofungin (Figure 1B). In *C. albicans*, *RFG1* is a regulator of filamentation, and has not previously been associated with caspofungin sensitivity [3,4]. Figure 1B also shows the effect of deleting *RBF1* and *RPN4* in *C. parapsilosis*, omitted from the species comparison because the deletions have a severe growth defect in *C. albicans*. Deleting *RBF1* reduces growth of *C. parapsilosis* in several conditions, whereas the most pronounced effect of deleting *RPN4* is sensitivity to ketoconazole (Figure 1B). Deleting *C. parapsilosis* *TUP1* has pleiotropic effects, as has also been reported for *C. albicans* [1].

We also determined the phenotypes of knockouts of 13 *C. parapsilosis* protein kinase genes (an additional three *VPS34*, *MSS2* and *YCK2*, have strong growth defects on YPD, and were not included). Two knockouts, (*CLA4* and *KIS1*), displayed highly pleiotropic phenotypes with restricted growth under many different conditions (Table S2, Figure 1B). Knocking out *MKC1* and *TPK2* conferred sensitivity to caspofungin, which has previously been reported for *C. albicans* [5]. Deleting several kinases (*CLA4, KIS1, MKC1, SIP3* and *TPK2*) reduces growth on media containing high levels of copper, but only *KIS1* was sensitive to lower copper concentrations.

*Comparison of copper and iron regulation in C. parapsilosis and C. albicans*

Regulation of copper sensitivity is also similar in the two species. Deleting the major copper-binding transcription factor*, CUP2* [1,6], results in severe growth defects of both species on copper-containing medium (Figure 1C)*.* Deleting *SFU1* which is part of the iron homeostasis regulatory circuit in *C. albicans* [6] results in a similar phenotype (Figure 1C). Deleting *FGR15* and *GZF3* confers sensitivity to copper stress in *C. albicans*. The *C. parapsilosis fgr15* and *gzf3* deletions form dark brown colonies (on media containing low concentrations of copper) suggesting that they also play a role in copper utilization in this species (not shown). In addition, the *C. parapsilosis fgr15* deletion exhibits a weak reduction in growth at very high concentrations of copper (Figure 1C, Table S2). Deleting *SEF2, SKO1,* and *RIM101* confers sensitivity to copper in *C. albicans* but not in *C. parapsilosis* (Figure 1C, Table S2).

The response to copper is closely related to the iron response [7]. Deleting many of the members of the *C. albicans* iron regulatory circuit (*SEF1, HAP2, HAP3, HAP5, HAP43*) [6] also reduces growth of *C. parapsilosis* in low iron conditions (Figure 1C, Table S2). Deleting *SEF2,* which is regulated by both Sef1 and Hap43 in *C. albicans* [6], increases sensitivity to copper in *C. albicans* but not in *C. parapsilosis* (Figure 1C). *SEF2* is a paralog of *SEF1* and is present in all CTG cade species, but not in *S. cerevisiae*. *SEF1* and *SEF2* are important for conferring resistance to caspofungin in *C. parapsilosis* but not in *C. albicans* (Figure 1C, Table S2). Deleting *SFU1*, one of the core iron regulators in *C. albicans* [6] does not noticeably affect growth of either species on low iron (Figure 1C). Expression of *CpSFU1* is increased when iron levels are low [8], but expression of the *C. albicans* ortholog is unaffected by iron levels [9]. There may therefore be subtle differences in the response to copper and iron in the two species.

1. Homann OR, Dea J, Noble SM, Johnson AD (2009) A phenotypic profile of the *Candida albicans* regulatory network. PLoS Genet 5: e1000783.

2. Uhl MA, Biery M, Craig N, Johnson AD (2003) Haploinsufficiency-based large-scale forward genetic analysis of filamentous growth in the diploid human fungal pathogen *C.albicans*. EMBO J 22: 2668-2678.

3. Kadosh D, Johnson AD (2005) Induction of the *Candida albicans* filamentous growth program by relief of transcriptional repression: a genome-wide analysis. Mol Biol Cell 16: 2903-2912.

4. Khalaf RA, Zitomer RS (2001) The DNA binding protein Rfg1 is a repressor of filamentation in *Candida albicans*. Genetics 157: 1503-1512.

5. Blankenship JR, Fanning S, Hamaker JJ, Mitchell AP (2010) An extensive circuitry for cell wall regulation in *Candida albicans*. PLoS Pathog 6: e1000752.

6. Chen C, Pande K, French SD, Tuch BB, Noble SM (2011) An iron homeostasis regulatory circuit with reciprocal roles in *Candida albicans* commensalism and pathogenesis. Cell Host Microbe 10: 118-135.

7. Philpott CC, Protchenko O (2008) Response to iron deprivation in *Saccharomyces cerevisiae*. Eukaryot Cell 7: 20-27.

8. Ding C, Vidanes GM, Maguire SL, Guida A, Synnott JM, et al. (2011) Conserved and divergent roles of Bcr1 and CFEM proteins in *Candida parapsilosis* and *Candida albicans*. PLoS ONE 6: e28151.

9. Lan CY, Rodarte G, Murillo LA, Jones T, Davis RW, et al. (2004) Regulatory networks affected by iron availability in *Candida albicans*. Mol Microbiol 53: 1451-1469.
